# Fermentation—A Potential Game Changer in the Development of Plant-Based Cream Cheese Alternatives

**DOI:** 10.3390/foods15152692

**Published:** 2026-07-30

**Authors:** Sophie Libberecht, Julia Matysek, Robert Sevenich, Cornelia Rauh, Myriam Loeffler

**Affiliations:** 1 Leuven Food Science and Nutrition Research Centre, Department of Microbial and Molecular Systems, KU Leuven, Campus Ghent, Belgium, Meat Technology & Science of Protein-Rich Foods (MTSP), 9000 Ghent, Belgium; 2 Department of Food Biotechnology and Food Process Engineering, Technical University Berlin, 14195 Berlin, Germany; j.matysek@tu-berlin.de (J.M.); r.sevenich@tu-berlin.de (R.S.); cornelia.rauh@tu-berlin.de (C.R.)

**Keywords:** fermentation, microbial exopolysaccharides, texture modulation, pulsed electric fields, ultrasound

## Abstract

Plant-based cream cheese alternatives are gaining popularity, yet replicating the sensory and nutritional attributes of dairy cream cheese remains challenging. Plant proteins often impart undesirable flavors and differ structurally from dairy proteins, resulting in weaker emulsifying and gelling properties, as well as compromised product structure and texture. Hydrocolloids are commonly used to improve organoleptic properties and/or spreadability but may introduce gummy textures and conflict with clean-label expectations. Fermentation represents a promising strategy to address these challenges. Exopolysaccharide (EPS)-producing starter cultures can naturally enhance texture. Depending on EPS type, concentration, molecular structure, and interactions with the food matrix, fermentation can improve creaminess, viscosity, and water-holding capacity while supporting cleaner-label formulations. In addition, fermentation may improve flavor, protein digestibility, and amino acid bioavailability and reduce antinutritional factors, while EPS may confer health-related benefits. This review examines how fermentation, particularly EPS biosynthesis, can improve the overall quality and texture of plant-based cream cheese alternatives, considering current market formulations. The reviewed literature indicates that fermentation and in situ-formed EPS offer potential, although their effects depend on strain selection, matrix composition, and processing conditions. Pulsed electric fields and ultrasound may enhance EPS production, representing an underexplored field of research. Future studies should assess these strategies in complex plant-based matrices to clarify matrix-specific effects.

## 1. Introduction

By 2050, the world’s population is expected to reach 9.7 billion people, accompanied by an increased demand for protein [1]. To ensure adequate protein supply, a dietary transition toward plant-based sources is necessary. Consumer demand for plant-based alternatives in Western countries is increasing, as reflected by the growing number of individuals following a vegetarian or vegan die, or identifying as flexitarians [2]. Primary drivers of this trend include health, environmental, and animal welfare concerns. The global plant-based food market is forecasted to grow from 29.4 billion U.S. dollars in 2020 to 161.9 billion U.S. dollars by 2030 [3]. However, plant-based dairy analogs still present many challenges regarding their nutritional profile, texture, and taste, which are the most critical determinants of consumer purchase decisions (besides product price) [4]. Overcoming these limitations would not only improve the nutritional and organoleptic quality of the products, but would also make them more attractive to flexitarians, who actively choose between animal protein- and plant protein-based products. This would accelerate the protein transition, highlighting the increasing importance and potential of research into plant-based foods.

Alongside vegan yogurt alternatives, plant-based cream cheese-type analogs (henceforth referred to as plant-based cream cheese alternatives) have gained popularity in recent years; however, mimicking the nutritional value and sensory experience of traditional cream cheese remains technologically challenging. Plant proteins often carry undesirable notes such as beany, grassy, or earthy flavors, depending on the botanical source utilized [5]. Furthermore, the resulting cream cheese alternatives are often directly acidified with organic acids such as citric acid, which fails to create (or mimic) the complex flavor profile introduced during microbial fermentation. Texture also remains an area of development in plant-based cream cheese alternatives, as the characteristic smoothness and spreadability of conventional dairy cream cheese-type products arise from specific colloidal interactions between casein micelles and milk fat globules. Together, these components form a cohesive emulsion gel with a fine microstructure, contributing to the product’s creamy mouthfeel and structural integrity [6]. In contrast, plant-based matrices are typically formulated using non-dairy proteins derived from sources such as legumes, nuts, oilseeds, and (pseudo)cereals. These proteins differ remarkably from animal proteins, such as caseins, in terms of molecular structure, solubility, and gelation behavior [7,8]. As a result, they often exhibit inferior emulsifying, gelling, and water-binding capacities, which can lead to less stable products and/or products with grainy mouthfeel. To address this, manufacturers frequently incorporate hydrocolloids [9]. While these additives can enhance viscosity and improve mouthfeel, they may also impart a gummy texture and are often perceived negatively by consumers seeking clean(er)-label products. Although the term “clean label” is not strictly defined, it generally refers to the absence of ingredients that consumers may perceive negatively, such as additives or chemical-sounding ingredients, and is commonly associated with short and easily understandable ingredient lists, a topic addressed in more detail by Asioli, et al. [10] in the review article “Making sense of the clean label trend”. Moreover, a cleaner label is often perceived as indicative of healthier and/or more natural products. A consumer survey involving 751 participants highlighted the positive perception of fermented products, such as yogurt, with consumers commonly associating these products with health benefits [11]. While Noguerol, et al. [12] showed that different consumer groups of plant-based alternatives, including flexitarians, vegans, vegetarians, and omnivores, appear to assign varying levels of importance to clean-label attributes, Appiani, et al. [13] systematically reviewed the consumer acceptance and sensory properties of plant-based analogs including dairy alternatives, also highlighting the central role of sensory characteristics including texture.

A promising approach to addressing the current challenges in plant-based dairy alternatives is to integrate a controlled fermentation step during processing. Fermentation with suitable starter cultures can reduce undesirable flavors and contribute to overall flavor perception through the formation of new aroma compounds [14,15]. Particularly through the use of exopolysaccharide (EPS)-producing starter cultures, fermentation may also contribute to the structure, and hence texture formation, of dairy analogs, as EPS exhibit functional characteristics similar to those of conventional hydrocolloids [16]. When produced in sufficient quantities, EPS can contribute to creaminess, viscosity, and water-holding capacity, offering a natural and especially label-friendly alternative to the above-mentioned additives [17]. Furthermore, fermentation can improve protein digestibility, increase amino acid bioavailability, and degrade antinutritional factors such as phytic acid and protease inhibitors, thereby promoting better protein quality and metabolic utilization [18]. More recent research highlights the health-promoting effects of in situ-produced EPS, further contributing to the nutritional profile of plant-based dairy alternatives [19,20]. The microbial in situ production of EPS is often a stress-stimulated process, as the biopolymers are formed as protective barriers against e.g., unfavorable environmental conditions, which offers possibilities to promote their formation [21,22].

This review aims to provide a comprehensive overview of the current formulation challenges in plant-based cream cheese alternatives and explores how fermentation can contribute to their mitigation. To this end, the review contains a market analysis of plant-based cream cheese-type alternatives currently available across the Western European market (complemented by additional screening of selected products from other European countries), focusing on key aspects such as nutritional composition, protein and fat sources, and the types of hydrocolloids (incl. thickeners) employed. The discussion then transitions to the potential of fermentation to enhance both the nutritional and the sensory attributes of dairy analogs, with a particular emphasis on the biosynthesis and functional role of EPS, and on strategies to enhance EPS production using emerging technologies such as pulsed electric fields (PEF) and ultrasound (US). While these technologies are typically applied for microbial inactivation in food processing [23,24], this review focuses on their application at sub-lethal levels, where they induce stress responses that influence metabolic activity and may thereby stimulate EPS biosynthesis, an emerging area of research that has not yet been comprehensively addressed in the scientific literature. The methodology underlying the review and market study is outlined in Appendix A.

## 2. Dairy Products and Plant-Based Cream Cheese Alternatives (Spreads)

Dairy products are an important source of high-quality, complete proteins, calcium, and vitamins including *B12* (cobalamin), *B2* (riboflavin), while also providing smaller amounts of vitamin D [25,26]. Calcium and vitamin D are critical for ensuring optimal bone mineralization and skeletal health. Fermentation has a long-standing tradition in the manufacture of dairy products, typically using lactic acid bacteria (LAB) such as *Streptococcus thermophilus* (often in combination with other starter cultures like *Lactobacillus delbrueckii* subsp. *bulgaricus* in yogurt products) to convert the milk sugar lactose into lactic acid, thereby influencing the final product’s texture, taste, digestibility, and microbial stability. The health-promoting effects linked to fermented dairy products containing probiotic strains such as *Lactobacillus acidophilus* LA-5 [27] or *Bifidobacterium* [28] are diverse, though they have primarily been attributed to positive effects on gut and immune health [26,29]. Despite the aforementioned nutritional benefits, dairy products also present challenges. Lactase non-persistence, lactose malabsorption, and lactose intolerance are common worldwide but should be distinguished from each other, as prevalence estimates vary markedly among populations, regions, age groups, and diagnostic methods [30]. Cow’s milk protein allergy in children [31], alongside a growing consumer preference for diets containing less saturated fat and a higher fiber content, are additional health-related factors driving the demand for dairy-free alternatives. Beyond health concerns, ethical considerations and environmental sustainability concerns are accelerating the shift toward plant-based alternatives to dairy products [32].

Cream cheese represents a significant segment of the global dairy product market, with a projected market size of around 9.1 billion U.S. dollars by 2025 [33], highlighting its relevance for industry, retail, and consumers. The growing interest in offering plant-based alternatives to dairy cream cheese is reflected in the increasing variety of spreadable vegan options available in retail. In 2024, the European plant-based cream cheese market was valued at 71.2 million U.S. dollars and is expected to nearly double by 2033. North America currently leads the plant-based cream cheese alternative market (89.4 million U.S. dollars in 2024), while the Asia-Pacific region shows the fastest market growth in this segment [34].

Despite the rising popularity of vegan (cream) cheese alternatives, challenges remain, particularly in achieving the complex organoleptic properties (texture and taste) and nutritional profiles that are comparable to their dairy counterparts. While the production of dairy cream cheeses follows a long tradition, plant-based alternatives have only emerged recently and usually consist of plant proteins, fats (oils), starches, and/or other functional ingredients that serve, e.g., as texturizing and stabilizing agents, as well as acidulants [35,36]. In general, two production routes exist: the fractionation route, which involves blending isolated plant proteins, water, starches, and/or other hydrocolloids with fats into a defined emulsion that is subsequently structured into a semi-solid matrix; and the tissue disruption route, in which less-refined or even unrefined ingredients, such as nuts or seeds, are physically disrupted to create a matrix composed of proteins, polysaccharides (e.g., starch), and lipids [7,36]. To establish a comprehensive understanding of the current market landscape and product composition, we conducted a market study prior to the literature review. This analysis examined commercially available plant-based cream cheese alternatives across the Western European market with additional screening of selected other European countries, including Austria, Belgium, Denmark, Finland, France, Germany, Greece, Ireland, Italy, and the Netherlands, revealing key formulation trends and compositional characteristics. We conducted this market study to bridge the literature review with actual product data, providing a more comprehensive understanding of the challenges and opportunities in the development of plant-based cream cheese alternatives through fermentation, as further discussed in Section 3. A total of 201 products from 65 different brands were analyzed with respect to their composition, including the types of protein and fat sources used, the application of hydrocolloids, and whether the products had undergone fermentation. The subsequent sections examine the ingredients commonly used in plant-based cream cheese formulations, addressing their sources and functional roles. These discussions are then linked to the data obtained from the market study to contextualize current formulation practices.

### 2.1. Plant Proteins Used in Plant-Based Cream Cheese Alternative Manufacture

**Figure 1:**
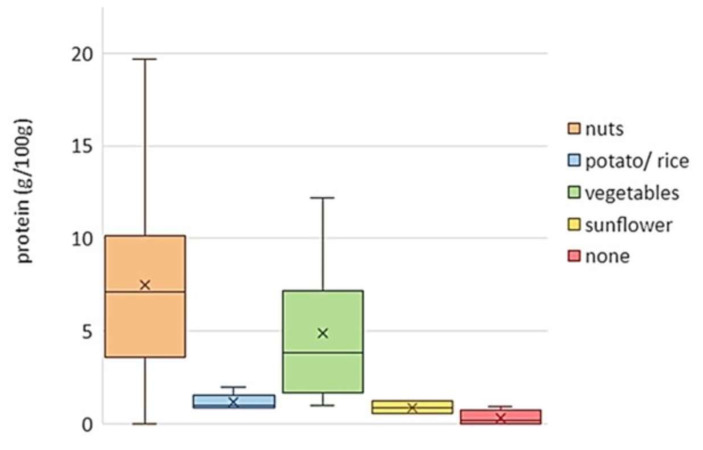
Average protein content per 100 g of plant-based cream cheese alternative, categorized by primary protein source (65 different brands were taken into account).

Plant proteins represent a promising resource for developing dairy alternatives such as milk substitutes, yogurt analogs, and vegan cream cheese alternatives [37]. In contrast to caseins, which are rheomorphic, intrinsically disordered proteins with well-defined techno-functional properties [38], most plant proteins currently used in food applications are storage proteins with predominantly globular structures, resulting in distinct structural organization and functional behavior. Protein quality is determined by amino acid composition, digestibility, and bioavailability. While animal proteins are generally complete and highly digestible, many plant proteins are limited in one or more essential amino acids, exhibit reduced digestibility due to antinutritional factors, and may impart off-flavors such as beany or grassy notes [39,40,41], posing challenges that are addressed in the following sections. Across the 65 brands analyzed in the market study, a variety of plant protein sources were incorporated into product formulations. The liquid phase of these products predominantly consisted of water or plant-based alternatives such as almond milk, cashew milk, coconut milk, or soy milk/juice. The majority of alternatives contained cashews (37.7%), almonds (20.3%), or soy protein (18.8%) as the primary protein source. Smaller proportions included potato (4.3%), rice (2.9%), sunflower (2.9%), and other sources such as oat, chickpea, faba bean, lentil, or pea protein (each <1.5%). Notably, 5.8% of the brands offered products without the addition of any plant-based proteins, while some brands utilized combinations of plant-based protein sources. Depending on the product formulation, the proteins were utilized as mentioned above or incorporated in the form of concentrates or isolates. The average protein content of the final products varied depending on the protein source and the specific brand or product. Figure 1 provides an overview of the average protein content per 100 g of product, categorized by primary protein source (e.g., nuts (including almonds), potato or rice, sunflower, and other vegetable proteins (e.g., lentil or pea)), and notable variations were observed across the different groups.

Soy protein has long been used in food products due to its techno-functional properties and high protein quality, with Protein Digestibility Corrected Amino Acid Score (PDCAAS) values that can approach those of dairy proteins [42]. However, soy also presents certain drawbacks, particularly related to its allergenic potential and limited local availability [43]. These limitations have contributed to a growing interest in exploring alternative plant proteins for the development of dairy analogs. Except for soybeans, most pulses are relatively high in starch and other carbohydrates, which can influence both their nutritional value and their functional properties. To achieve nutritional profiles that more closely resemble those of animal-derived products, processing steps such as concentration and isolation are often employed to obtain functional ingredients with a high protein content [44]. The amino acid composition of proteins used in plant-based dairy alternatives also varies depending on the cultivar, environmental conditions, and processing history, including extraction and precipitation techniques [45]. Pea protein is frequently used in vegan dairy analogs (though still only on a limited basis in plant-based cream cheese alternatives) because of its protein quality, techno-functionality, including gelling and emulsifying properties, and low allergenicity. Pea protein consists of two main fractions: globulins (major salt-soluble fraction) and albumins (water-soluble fraction). The globulin fraction includes storage proteins such as legumin (11S), vicilin (7S), and convicilin (8S), each differing in subunit composition, which in turn influences their techno-functional properties [46]. While pea protein contains particularly high levels of the essential amino acids lysine, (iso)leucine, and valine, the concentration of methionine, cysteine, and tryptophan is rather low, with methionine being the primary limiting amino acid. Other legumes relevant to vegan dairy alternatives include faba bean, lupin, and chickpea proteins [35], all of which exhibit a moderately balanced Essential Amino Acid Index (EAAI). These proteins are particularly rich in lysine but limited in (essential) sulfur-containing amino acids [47]. They demonstrate specific techno-functional properties, including emulsifying, gelling, and foaming capacities, as reviewed by other authors for lupin [48], faba bean [49], and chickpea protein [50]. Moreover, when examining the raw materials themselves, they all serve as substantial sources of dietary fiber. For instance, whole faba beans can contain up to 30% total dietary fiber, with (hemi)cellulose and lignin being the primary components [51]. Legumes are also sources of micronutrients such as iron and calcium, as well as of several B vitamins, with the exception of cobalamin (vitamin *B12*), which is generally absent from unfortified plant-based foods [35,52]. Lately, the use of sunflower and pumpkin seeds has become more popular. While both are limited in lysine, pumpkin seed protein has a high content of hydrophobic amino acids (35.67 mol%), influencing its techno-functional properties (e.g., gelation and water retention) [53,54]. Moreover, sunflower seeds are a valuable source of vitamin E and contain notable amounts of oleic and linoleic acids [55], whereas pumpkin seeds are recognized for their zinc content [56]. All seeds and legumes may pose allergen risks. However, while these risks have been reported to be very low for many sources, such as pea protein or the oilseeds mentioned, lupin has been specifically identified as an allergen under EU labeling legislation [57]. In addition to pulses, nut-based proteins are increasingly utilized in dairy analogs, with their amino acid spectra and bioavailability varying depending upon the source and processing method used. House, et al. [58] reported PDCAAS values for raw almonds of 44.3–47.8 (on a 0–100 scale) and confirmed lysine to be the limiting amino acid. Although coconut proteins play an important role in the functionality and emulsion stability of coconut milk [59], whole coconut flesh contains only about 3–4% protein, making it a minor protein contributor in formulations. Therefore, coconut is usually not applied as a concentrate/isolate, but in the form of milk or cream, and the products/ingredients are primarily valued for their fat content, creaminess, and taste. Other protein sources identified in the market analysis include potato, hemp, and the cereals oat and rice. All of these protein sources differ in their amino acid composition, influencing both nutritional quality and functionality. Potato protein stands out with regard to its high lysine content and high PDCAAS and is known for its great water solubility, gel-forming capacity, and emulsifying properties [60,61]. While lysine is a limiting amino acid in rice protein, methionine and leucine are more abundant. Hemp protein has a relatively balanced essential amino acid profile except for lysine, which is limited [47]. This is also the case for oat protein, which has been proposed as a suitable alternative for use in dairy analog production [62]. Improved PDCAAS or DIAAS (Digestible Indispensable Amino Acid Score) values can be achieved when lysine-poor cereals like oats are blended with lysine-rich proteins, as has been reported for various plant-based protein combinations [63]. To achieve complete amino acid coverage and improve protein quality, several strategies have been developed, as further discussed in Section 3.

### 2.2. Fat Sources Used in Plant-Based Cream Cheese Production

Fat plays a crucial role in the structure, texture, sensory perception, and nutritional quality of plant-based cream cheese alternatives, with its functionality being strongly influenced by fatty acid composition. For instance, coconut and palm fat are high in saturated fatty acids and are widely utilized due to their ability to provide solid fat at ambient temperatures [64], thereby contributing to the firmness and spreadability required to mimic conventional dairy cream cheese. In contrast, oils rich in unsaturated fatty acids, including sunflower, rapeseed (canola), and corn oil, are often incorporated to improve the fatty acid profile and enhance the nutritional quality of the products [65]. Consequently, the formulation of fat blends combining saturated and unsaturated lipid sources seems to be an emerging strategy in plant-based cream cheese alternative production. For further in-depth information on fats used in non-dairy food applications, readers are referred to Ray, et al. [66].

**Figure 2:**
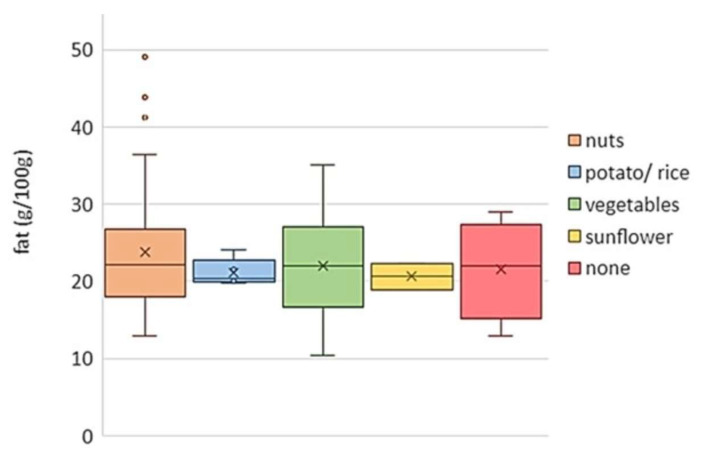
Average fat content per 100 g of plant-based cream cheese alternative, categorized by primary protein source (65 different brands were taken into account).

Our market analysis revealed variability not only in the types of fat sources used across the products examined, but also in their fat content, with several brands offering reduced-fat formulations (Figure 2). In the majority of products (>50%), coconut oil served as the primary fat source, followed by rapeseed oil, sunflower oil, and olive oil. A limited number of formulations also contained linseed oil or coconut fat/butter. Palm oil, as well as specialty oils such as walnut, shea, and truffle oil, were identified in only one or two products across the entire product range analyzed in the market study.

### 2.3. Hydrocolloids

As previously noted, plant proteins exhibit functional properties that differ substantially from those of their dairy counterparts, which often necessitates the incorporation of food hydrocolloids to enhance textural characteristics and better replicate the structural and organoleptic attributes of dairy cream cheese. Hydrocolloids are a heterogeneous group of long-chain polymers, primarily polysaccharides and, to a lesser extent, some proteins (e.g., gelatin) that are derived from a variety of natural sources including plants, microorganisms, and animals, as well as naturally occurring polysaccharides that have been chemically modified (e.g., carboxymethylcellulose) [67]. Hydrocolloids are widely utilized in the food industry due to their diverse functional properties, including thickening, gelling, foam and emulsion stabilization, controlled flavor release, and the inhibition of ice crystal formation, and thus contribute to the structure, texture, and organoleptic properties of food products [68,69,70]. At the molecular level, hydrocolloids typically exhibit a high density of hydroxyl groups, which imparts strong hydrophilicity and enables extensive water-binding interactions [70]. Their molecular configuration and polymeric characteristics influence hydration behavior and determine their functionality as gelling, thickening, and stabilizing agents in food systems. Thickening refers to an increase in viscosity resulting from polymer hydration and chain expansion in solutions. In this case, no (true) permanent or semi-permanent three-dimensional gel network is formed [70]. Gelling, in contrast, involves the formation of a continuous, three-dimensional network that traps water and other components, resulting in a solid-like or semi-solid structure. In this case, polymer chains interact via junction zones, commonly stabilized by hydrogen bonding, ionic interactions, or crystallization processes [70,71]. From a rheological perspective, a hydrocolloid gel is defined as a viscoelastic system in which the storage modulus (G′), representing the elastic or solid-like behavior, exceeds the loss modulus (G″), which represents the viscous or liquid-like behavior [71,72]. A strong gel is characterized by G′ ≫ G″, indicating dominant elastic behavior, whereas a weak gel exhibits G′ > G″, reflecting a more fragile network [72]. Hydrocolloid gels can be thermo-reversible, where gelation occurs upon cooling (e.g., agar) or upon heating (e.g., methylcellulose) and reverts to a sol state upon temperature reversal [73]. In contrast, non-thermo-reversible gels arise from ionic or covalent cross-linking mechanisms. An example is the Ca^2+^-mediated gelation of alginate, forming a stable network that does not melt upon heating but may be disrupted by chelating agents. Examples of hydrocolloids with strong gelling properties include agar, alginate, carrageenan, pectin, gellan gum, and (outside the vegan context) gelatin [74]. Hydrocolloids primarily used for their thickening effects include gum arabic (acacia gum), xanthan gum, galactomannans (e.g., locust bean gum and guar gum), cellulose derivatives, and native or modified starches [70]. While native starches gelatinize upon heating, thickening the system through granule swelling and amylose leaching, high-amylose starches can form true gels upon cooling due to amylose retrogradation. Modified starches can be tailored to behave more as thickening agents (e.g., pregelatinized starch) or more as gelling agents (e.g., cross-linked starches) [75,76]. Moreover, several other hydrocolloids, including carrageenan or pectin, are also multifunctional, meaning that they can act as thickeners at sub-gel levels and form gels under suitable conditions. Beyond thickening and gelling, hydrocolloids also play a key role in stabilizing emulsions and suspensions.

**Figure 3:**
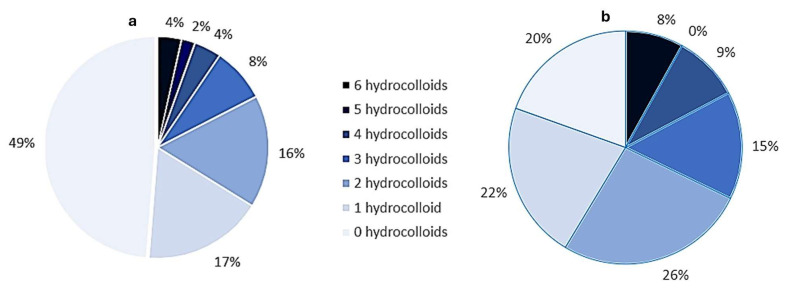
Number of different hydrocolloids being used in plant-based cream cheese alternatives (based on our own European market study—201 products from 65 brands). (**a**) Data from all 201 products and (**b**) data from products formulated with protein sources other than nuts and almonds (*n* = 87).

In plant-based dairy alternatives, hydrocolloids including (modified) starches are utilized to promote product stability while providing spreadability, and a pleasant texture and mouthfeel. Due to their different functionalities, combinations of hydrocolloids are often utilized for their synergistic effects [76], as also reflected in the conducted market study. To interpret the results related to hydrocolloids, two analytical approaches were applied. Figure 3a presents data from all 201 products analyzed, highlighting the number of different hydrocolloids being used. Notably, products based on nuts, particularly due to their specific formulation characteristics (typically via the “plant tissue disruption method”), were often found to contain no added hydrocolloids. However, some of these products included ingredients such as citrus fiber or inulin, which, although not classified as hydrocolloids in the present analysis, are known for their pronounced water-binding capacity. In a second approach, products formulated with protein sources other than nuts (incl. almonds) were examined separately, as the use of hydrocolloids was considerably more prevalent (Figure 3b). Here, often more than one (up to six) different hydrocolloids have been applied in order to improve the texture and mouthfeel characteristics of the products while promoting product stability. In this regard, the top six hydrocolloids that have been used include (modified) starch from sources such as maize/corn, potato, tapioca, guar gum/flour, xanthan gum, locust beam gum, carrageenan, or agar agar.

The concentration and ratio of the added hydrocolloids were highly product-dependent. While hydrocolloids are widely employed to improve texture and stability, their use can also present technological challenges. At elevated concentrations or in certain combinations, hydrocolloids may increase gel brittleness or interfere with protein network formation, as e.g., demonstrated in low-fat cheddar cheese [77]. Such destabilization effects can compromise structural integrity and lead to phase separation. Starch-based thickeners, commonly used for viscosity enhancement, are particularly susceptible to retrogradation during storage, resulting in undesirable textural changes such as syneresis and increased hardness [78]. In addition to technical limitations, hydrocolloids are frequently associated with negative consumer perceptions related to ingredient labeling, which has intensified the demand for clean-label alternatives.

### 2.4. Other Ingredients and Link to Fermentation (Market Study)

On average, the products screened in the market study contained approximately 1% salt. Additionally, 28.6% of the products contained added sugars, primarily in powdered form (e.g., raw cane sugar), and to a much lesser extent in the form of syrups, including those derived from glucose, rice, or agave. Some formulations contained preservatives such as sorbic acid (E200) or potassium sorbate (E202), and in a limited number of cases, antioxidants were also listed on the ingredient label. Furthermore, emulsifiers were listed for only a few products. Depending on the product formulation, various herbs and spices were used, and several products additionally contained flavor-enhancing ingredients such as yeast extract or nutritional yeast. As previously noted, the majority of commercially available plant-based cream cheese alternatives are not fermented. Instead, acidity is typically adjusted through the addition of organic acids, with lemon juice (concentrate or extract) and citric acid being the most commonly used acidulants in the products investigated. These were followed by lactic acid/sodium lactate and vinegar derived from various sources (e.g., grapes or apple cider). Approximately 30% of the products analyzed were either fermented themselves or contained at least one fermented ingredient, such as tofu or other fermented vegetables. This finding indicates that truly fermented products currently represent only a minor segment of the plant-based cream cheese market. However, the presence of fermented ingredients or of starter cultures in a subset of products suggests that certain manufacturers are increasingly exploring fermentation-based strategies to enhance the sensory, technological, and overall quality characteristics of their products.

## 3. Fermentation and the Potential of EPS-Producing Starter Cultures

While a wide variety of plant-based cream cheese alternatives are already available on the market, challenges persist, particularly in achieving enhanced texture, taste, and nutritional quality, alongside the growing consumer demand for cleaner-label formulations. In this context, fermentation represents a promising strategy to address these limitations, as a technology that is well-established in dairy product manufacture, where EPS-producing strains are commonly employed in order to improve mouthfeel and/or reduce the likelihood of syneresis during storage. As described in more detail in Section 2.1, plant proteins typically exhibit a promising but often less balanced amino acid profile, often being limited in sulfur-containing amino acids such as methionine and cysteine in legumes or lysine in cereals [47]. Moreover, while being a valuable source of minerals and vitamins, the digestion and subsequent absorption, and thus the bioavailability, of proteins and micronutrients are impaired by antinutritional factors such as phytates, tannins, lectins, and protease inhibitors, which are abundant in legumes and oilseeds [79]. For instance, phytates in soybeans or (chick)peas have been shown to chelate divalent minerals such as zinc and calcium, thereby reducing their bioavailability [80,81]. When ingested, tannins in e.g., legumes, cereals, and seeds were found to form complexes with proteins, leading to the inactivation of digestive enzymes and hence to reduced protein digestibility, which has been studied and reviewed by other authors [39,82]. Moreover, legumes are frequently associated with undesirable flavor notes, commonly described as “beany” or “grassy”, which are primarily attributed to lipid oxidation pathways including the formation of volatile oxidation products [83]. Bitterness and astringency, on the other hand, are linked to the presence of compounds such as alkaloids, isoflavones, and saponins. The intensity of the undesired flavors and bitter attributes can vary depending on the plant variety and the environmental conditions during growth. For instance, when linoleic acid and α-linolenic acid are present, their lipid oxidation products (specifically oxylipins) have been identified as key contributors to bitterness in pea protein isolates [84]. In canola protein isolates, a complex glycoside derivative of kaempferol has been recognized as the primary bitter compound [85]. As these challenges restrict the application potential of plant proteins in dairy (and meat) analogs, recent research efforts increasingly address them through interventions at both the raw material and processing levels. On the raw material side, strategies include the breeding and/or selection of e.g., legume varieties with reduced levels of antinutritional factors and “undesirable” flavor precursors [86] and the use of omics technologies [87]. Pretreatment technologies that have been reported to improve protein quality and reduce levels of antinutritional factors further include, but are not limited to, (i) germination [88], (ii) soaking and heating [89,90], (iii) enzymatic modification/hydrolysis [91,92], and/or fermentation [18,93,94], as well as (iv) advanced protein fractionation [95].

In order to achieve more complete amino acid coverage and improve overall protein quality, blending protein sources that are limited in methionine (legumes) with those that are limited in lysine (cereals) is another strategy. Blending may thereby not only improve the nutritional quality, but also the overall techno-functionality [96]. In addition to blending different proteins, enzymatic treatments and/or fermentation have been shown to improve the nutritional profile of plant proteins. For instance, Kim, et al. [97] showed that enzymatic modification and fermentation with LAB (*Lactiplantibacillus plantarum* CKDHC 0801 and *Levilactobacillus brevis* KCCM 11509) improved both the digestibility and the bioavailability of pea protein. This was also in accordance with a study performed by García Arteaga, et al. [98] in which enzymatic hydrolysis with proteolytic enzymes and fermentation with *L. plantarum* resulted in pea protein ingredients with better solubility and functionality. In addition, the authors further reported improved sensory characteristics, as the combined treatment resulted in a reduction of undesired flavor notes. Fermentation has also been shown to be a suitable “tool” for the debittering of plant protein hydrolysates and/or for improving the overall aroma profile of plant proteins (isolates) including for example pea [99,100,101], soy [102], or lupin [103]. Moreover, targeted co-fermentation (with e.g., *Propionibacterium freudenreichii* [104]) can serve as an effective strategy for the production of vitamin *B12*, which is naturally absent in plant protein-based products that are hence often fortified to obtain a nutritional profile similar to that of their dairy counterparts [105,106]. The already-mentioned health-promoting effects associated with fermented dairy products that contain probiotic strains [28] may also apply to plant-based alternatives. In addition, the EPS formed by LAB are not only known for their texture-modifying properties, but are also associated with prebiotic effects [107,108,109], with prebiotics being substrates that are selectively utilized by host microorganisms, conferring a health benefit [110]. For instance, EPS from LAB have been reported to exhibit immunomodulating, anti-cancer, and antiviral properties, as reviewed by other authors [19,111].

As highlighted in Section 2.1, the functional properties of plant proteins are usually less pronounced than those of dairy proteins. Many plant proteins, particularly legume- and pulse-derived proteins, are rich in globulins and albumins with compact conformations, often showing reduced solubility, weaker emulsifying capacity, and limited gelation ability, which constrains their performance in vegan cream cheese alternatives [8,112]. Product structure, and hence texture attributes, are therefore often obtained by combining plant proteins with hydrocolloids including e.g., (modified) starches and/or carrageenan, among others (see Section 2.3) [113]. These compounds not only enhance organoleptic properties, but also influence the nutritional profile of the food products and contribute to longer ingredient lists, which is often negatively perceived by consumers due to labeling requirements. While fermentation has already been introduced as a powerful strategy to address challenges related to nutritional quality and flavor, its potential role in texture modification warrants further attention. While EPS-producing starter cultures have a well-established history in the dairy industry, their application in plant-based dairy alternatives remains a relatively new and emerging area of research. Likewise, the use of novel non-thermal technologies such as pulsed electric field (PEF) and ultrasound (US) to stimulate microbial EPS production is still in its early stages, offering promising avenues for innovation in texture enhancement and clean-label formulation. As a non-thermal preservation and intensification method, PEF is particularly advantageous for retaining heat-sensitive nutrients and preserving sensory qualities compared to conventional thermal treatments. In plant-based matrices, PEF has also been shown to influence the bio-accessibility of health-promoting phytochemicals, as demonstrated, for instance, by increased carotenoid levels in treated tomato products [114]. Moreover, González-Casado, et al. [115] reported increased in vitro bio-accessibility of tomato carotenoids (lycopene, lutein, and β-/γ-carotene), which can be attributed to electroporation-induced tissue disintegration [116]. In addition to PEF, power ultrasound has also been shown to preserve or enhance health-promoting quality attributes, such as phenolic content, antioxidant capacity, and enzymatic activity control. Unlike conventional thermal treatments, which often lead to nutrient degradation, power ultrasound can even promote the release of antioxidants and phenolic compounds in certain vegetables [117] and maintain or improve protein bio-accessibility in plant-protein/fiber systems, as reviewed by other authors across different food matrices [118,119,120]. While fermentation with EPS-forming starter cultures, PEF, and ultrasound have each gained attention, this review evaluates in more detail how PEF and ultrasound, when applied under appropriate conditions, may induce microbial stress responses that enhance in situ EPS synthesis. This, in turn, can contribute to texture development, potentially enabling product formulations with fewer added ingredients, particularly by reducing or eliminating hydrocolloids in the manufacture of dairy analogs. This review concludes with an overview of plant-based dairy analogs produced through fermentation, with a focus on vegan cream cheese alternatives. Given the limited literature on this topic, examples from plant-based yogurt production are also included to illustrate the potential of fermentation and EPS-producing LAB in addressing texture-related challenges in spreadable (spoonable) dairy analogs. Findings of this review part may also serve as a foundation for developing other plant-based matrices, broadening the application potential of fermentation and EPS-producing cultures.

### 3.1. Microbial Exopolysaccharides (EPS) and Their Biosynthesis

This review article focuses on EPS produced by LAB, which are known to have a functional role in the rheology and sensory profile of fermented dairy products [17,121]. In general, microbial polysaccharides can be broadly classified into internal and external types. Internal polysaccharides, often referred to as intracellular storage polysaccharides, are retained within the microbial cell and primarily serve as energy reserves. The external polysaccharides can be further divided into capsular polysaccharides (CPS), which remain closely associated with the cell surface, and exopolysaccharides (EPS), which are released into the surrounding environment and are less tightly associated with the cell [122,123]. Both CPS and EPS contribute to the formation of a glycocalyx, which can be described as a polymeric layer surrounding the cell surface [124]. EPS are defined as long-chain, high-molecular weight, linear or branched polysaccharides, which can differ considerably in their monosaccharide composition, molecular structure, and physicochemical characteristics [125,126]. These polymers have several functions such as cell recognition and interaction, biofilm formation, and surface adherence [121,126]. Further, EPS can be divided according to their composition into homopolysaccharides (HoPS) and heteropolysaccharides (HePS). LAB HoPS comprise polymers built from a single monosaccharide type, typically glucose (glucans), fructose (fructans), and in some cases galactose (galactans/polygalactans). Glucans are classified into α-glucans (dextran, mutan, alternan, and reuteran) and β-glucans (e.g., ropy β-glucan from *Pediococcus parvulus* [127]), fructans into inulin-type and levan; and galactans (polygalactans) form a separate HoPS category [128,129]. Many α-glucans and fructans are synthesized extracellularly from sucrose by glucansucrases or fructansucrases, whereas other polysaccharides, such as some LAB-derived β-glucans, are produced via membrane-associated glycosyltransferases using nucleotide-sugar precursors [127,130]. Recent studies also reported strain-specific bacterial synthesis of α-galactans from sucrose [131]. HePS are composed of neutral or charged repeating units, which typically consist of α- or β-linked di- or oligosaccharide structures made from various monosaccharides such as glucose, galactose, rhamnose, mannose, and occasionally uronic acids. In addition, HePS often contain non-carbohydrate substituents like phosphate or acetyl groups, which influence their charge and physicochemical properties [132,133,134]. Examples of well-known heteropolysaccharides include xanthan, gellan, and alginate, while kefiran is a well-established LAB-derived heteropolysaccharide [135]. The molecular mass of HoPS is generally higher (often >10^6^ Da) compared to HePS (10^4^–10^6^ Da). The production yield of HePS is lower than that of HoPS, typically reported in the mg/L range for HePS and in the g/L range for HoPS, which is attributed to the more energy-demanding biosynthesis [129,133] and the greater structural complexity of HePS [136]. Due to the latter, a lower amount of HePS is often sufficient to affect the properties of a certain food product. Several studies have shown that environmental conditions play a role in modulating the properties of the EPS formed, which in turn govern their functionality in food products. Zehir Şentürk, et al. [137] investigated the influence of several conditions (incubation temperature, time, pH, and carbon source) on the production, composition and viscosity of EPS produced by six *Lactobacillus plantarum* strains isolated from Tarhana and reported that EPS production levels, composition, and rheological properties were dependent on both strain and condition. Similarly, Minari, et al. [138] showed that the strain *L. casei* Ke8 was able to produce HePS in semi-defined media containing glycerol, glucose, or molasses as carbon sources. The HePS produced from glycerol and glucose consisted mainly of glucose and mannose and yielded 3.0 g/L. In contrast, when molasses was used as the substrate, galactose and arabinose were also incorporated into the EPS structure alongside glucose, which was associated with a different molar mass of the EPS and a slightly higher yield of 3.5 g/L. Moreover, Prechtl, et al. [139] showed that cold and salt stress, both of which are important parameters in meat production, modulated the amount and structure of EPS (dextran) produced by *Latilactobacillus sakei* 1.411. They reported that a temperature of 10 °C resulted in a higher production level, molecular weight, and particle size compared with 30 °C. In addition, high salt concentrations reduced EPS production and limited EPS, i.e., dextran polymerization. Furthermore, Zhang, et al. [140] investigated the effect of the molecular weight of EPS produced by *L. helveticus* on the gel properties of yogurt and reported that EPS with a higher molecular weight were associated with increased viscosity, improved water-holding capacity, and a denser and more homogeneous network. Gentès, et al. [141] reported that structural characteristics of EPS such as negative charge, flexibility, degree of branching, and molecular weight modify the gel formation and rheological properties of fermented milk produced with different EPS-producing strains. The authors found that the physical and rheological properties of set yogurt fermented by different EPS-producing strains (*Streptococcus thermophilus* HC15, 2104, NIZO 0131, and *Lactobacillus delbrueckii* subsp. *bulgaricus* 210R, ATCC 11842, NCIMB 702074, DGCC 291) were influenced by the structural characteristics of the EPS synthesized. Negatively charged EPS influenced gel formation and contributed to the elasticity of the network, while high molecular weight EPS with stiff chains and limited branching improved apparent viscosity, firmness, and whey retention. Together, these findings highlight that both environmental conditions and EPS functional properties contribute to the final characteristics of food products. This is particularly relevant because LAB-derived EPS can act as structure and texture modifiers, providing emulsifying and stabilizing properties and enhancing overall product texture [16,142,143]. Their role as texture modifiers will be subsequently discussed in more detail for dairy products and plant-based alternatives (Section 4).

The biosynthesis of EPS is a genetically and enzymatically complex process, with ongoing research focused on identifying the specific genes and regulatory mechanisms involved [144,145]. The general steps underlying HePS and HoPS biosynthesis have been comprehensively reviewed by Schmid, et al. [146]. The following four main EPS-synthesis pathways have been described in bacteria, including LAB: (i) the Wzx/Wzy-dependent pathway; (ii) the ATP-binding cassette (ABC) transporter-dependent pathway; (iii) the synthase-dependent pathway; and (iv) the extracellular synthesis via a sucrase-mediated mechanism. The first three pathways rely on intracellular sugar nucleotide precursors, whereas the sucrase-based route utilizes extracellular sucrose as the direct substrate. In the Wzy-dependent pathway predominant for LAB HePS, glycosyltransferases (GTs) assemble repeat units on a C55-undecaprenyl phosphate (Und-P) lipid carrier at the cytoplasmic membrane. Wzx flips the lipid-linked repeat unit across the cytoplasmic membrane, and Wzy polymerizes the repeats at the external face of the cytoplasmic membrane, after which the polymer is further exported through the cell wall, becoming cell-associated (capsular) and/or released into the surrounding matrix, depending on the strain and polymer [122,147]. Polysaccharides formed by the Wzx/Wzy-dependent pathway are generally heteropolymers, consisting of different types of monosaccharides [146]. The ABC transporter-dependent pathway also involves GT-mediated polysaccharide assembly but differs in the export mechanism [122,148]. This pathway is mainly associated with the biosynthesis of capsular polysaccharides and can yield either HoPS or HePS, depending on the substrate specificity and diversity/combination of GTs encoded within the operon. Notably, in well-studied Gram-negative bacteria, capsular polysaccharides synthesized via the ABC transporter-dependent pathway are distinguished from freely secreted EPS by the presence of a conserved glycolipid at the reducing end, promoting cell-surface association [148]. In synthase-dependent pathways, a membrane-associated processive synthase couples polymerization and translocation without Wzx/Wzy repeat-unit flipping; in LAB, this mechanism is exemplified by ropy β-glucan synthesized from UDP-glucose by a membrane-associated enzyme. In contrast, dextran and levan/inulin-type fructans are typically produced extracellularly from sucrose by glucansucrases and fructansucrases, respectively [146]. Accordingly, sucrose represents the primary donor substrate for extracellular sucrase-mediated HoPS synthesis in food matrices fermented with these strains, as energy is released upon cleavage of the glycosidic bond. By comparison, HoPS synthesis from free glucose or fructose relies on intracellular pathways that require the expenditure of ATP and/or UTP equivalents to generate activated sugar-nucleotide precursors prior to polymerization, as previously described [149,150]. However, yields obtained from these monosaccharides are often substantially lower than those achieved with sucrose. Although less relevant for industrial-scale applications in the food industry, this phenomenon remains of considerable scientific interest, as it suggests the existence of distinct enzymatic mechanisms and regulatory pathways that enable HoPS synthesis under sucrose-limited conditions. Such mechanisms may reflect adaptive strategies employed by LAB in specific ecological niches where carbohydrate availability is variable. Understanding these processes could provide valuable insights into carbohydrate metabolism, stress responses, and the diversity of EPS biosynthesis in LAB. Furthermore, such knowledge could inform future metabolic engineering approaches aimed at optimizing EPS production from diverse substrates. Despite this potential, current omics-based investigations have largely focused on sucrose-dependent HoPS biosynthesis, leaving monosaccharide-driven pathways for HoPS production as an underexplored area of research. In parallel, the mechanisms governing EPS production, particularly HePS, are still not fully understood. Recent research increasingly focuses on elucidating the underlying pathways by further investigating the enzymes and genes involved, employing omics-based approaches [151]. Consequently, more recent studies and reviews have emerged that provide novel insights into these biosynthetic processes [144].

Research on EPS-producing starter cultures has resulted in the development of diverse methodologies for investigating HePS and HoPS formed in food matrices under varying conditions. Prior to analysis, EPS must be isolated from the food matrix and subsequently purified. A range of analytical techniques is available to characterize EPS, including the determination of monosaccharide composition by e.g., HPLC [152], the determination of molecular weight (distribution), and the investigation of surface morphology using microscopic techniques such as scanning electron or atomic force microscopy [153], as well as further structural characterization through e.g., X-ray diffraction or Fourier transform infrared spectroscopy [154]. These approaches have substantially advanced the understanding of the functional roles of EPS in food systems. Nevertheless, direct comparisons across studies remain challenging, as parameters such as EPS yield and molecular weight can vary depending on the growth conditions and matrix composition, even when produced by the same microbial strain [139]. For further reading on the detection and characterization of EPS, the reader is referred to the review articles by Ruas-Madiedo and de los Reyes-Gavilán [125] and Yadav, et al. [155]; the latter also provides an overview of methods for determining thermal characteristics.

### 3.2. Stimulating EPS Production

Under stress conditions, microorganisms develop genetic, physiological, and metabolic responses. These changes can result in altered metabolic activity, including improved productivity and fermentation rates, reduced by-products, and/or the production of new compounds and/or higher yields [156]. The relationship between stress and microbial response is highly dependent on the specific microorganism and the intensity of the stress applied [23,157]. Under extreme stress, cells may be unable to adapt or survive, resulting in microbial inactivation or destruction. This principle is the basis of food pasteurization, where the goal is to eliminate vegetative microbial contaminants to ensure food safety and prolong preservation [158]. However, when mild stress (at sub-lethal levels) is applied, microorganisms can react by activating specific stress response mechanisms and potentially adapting to the new environment [159]. For some LAB, their stress response is the production of EPS [22,160] that are then secreted to the extracellular medium to protect the microorganisms from negative environmental conditions in the form of capsules attached to the cell wall or by forming a mucus layer around the cell [160,161], as described in Section 3.1. Various strategies have therefore been investigated to modulate microbial metabolism and stress responses by exposing microorganisms to controlled stress conditions. In addition to conventional thermal strategies of stressing microorganisms, other promising approaches involve non-conventional and non-thermal technologies, such as pulsed electric fields (PEF) and ultrasound (US). While these technologies are typically used for microbial inactivation, extraction, or tissue softening in food processing [23,24], this review highlights their use at sub-lethal levels to induce adaptive stress responses and modulate metabolic activity, including EPS production. While a variety of emerging technologies may influence fermentation processes, this review specifically focuses on PEF and US, as both have demonstrated strong potential to induce controlled microbial stress responses relevant to EPS production. These non-thermal technologies enable modulation of microbial metabolism while preserving heat-sensitive nutrients and sensory attributes, making them particularly promising for application in plant-based dairy alternatives. Other approaches, such as high-pressure processing, enzymatic treatments, or thermal methods, have also been explored in the context of food processing. However, these technologies are not discussed in detail here, as their mechanisms are less directly linked to targeted microbial stress induction and EPS biosynthesis.

The following sections will explore the mechanism of the aforementioned PEF and US, their impact on cellular stress responses, and the challenges associated with applying these technologies in fermentation processes.

#### 3.2.1. Pulsed Electric Fields (PEF)

##### Mechanism of the Technology

PEF is a non-thermal technology that can be used to process various foods or biomatter of different origins [162]. The treatment consists of the application of very short electric pulses (e.g., in the nanosecond, microsecond, or millisecond domains, yielding nsPEF, μsPEF, and msPEF treatments, respectively) at electric field intensities in the range of 0.1–40 kV/cm, applied by a pulse generator. Electric field (E) intensities can be differentiated as low (E < 2 kV/cm), moderate (E = 1–5 kV/cm), or high intensity (E = 10–40 kV/cm), with energy inputs around 0.1–150 kJ/kg. The field intensity leads to the formation of a critical transmembrane potential, which is regarded as the precondition for cell membrane breakdown and the phenomenon of electroporation [163,164]. In the simplest cell membrane model, the membrane consists of bilayer sheets formed by phospholipids, with their polar phosphate groups oriented toward the aqueous intracellular and extracellular environments. In biological cells, an intrinsic (resting) transmembrane potential of around 10 mV is typically generated [165]. However, an external electric field can induce an additional transmembrane potential. The charging and polarization of the membrane occur when the charging time exceeds 1 μs. Since the electrical conductivity of membranes is much lower than that of the surrounding aqueous solution, the external electric field primarily focuses on the non-conductive membranes and leads to electro-compression of the membrane through attraction of charge. When reaching the critical transmembrane potential, the development of pores, the so-called electroporation, occurs. This phenomenon of electroporation is influenced by cell size, spatial orientation, the electrophysical properties of the cells, the pH of the surrounding medium, and the presence of osmotic agents [162]. Changes in the electric field strength, the treatment temperature, the pulse characteristics, and the electrical properties of the suspended cells influence the temporal behavior and the extent of membrane permeabilization [166,167]. The phenomenon of membrane breakdown was first reported in 1957 [168]. Early studies observed both reversible and irreversible damage effects, depending on the intensity of the electric field strength applied. The stimulating effects of electricity on the growth of bacteria and yeast were documented as early as 1909 [169], where a significant increase in bacterial count (up to a 100-fold increase compared to the control group) was noted as a result of electrical stimulation with weak currents. Electroporation is a dynamic process and dependent on treatment parameters and the treated strain [170,171]. Depending on the treatment intensity (external electric field strength, number and duration of the electric pulses) and cell properties (size, shape, orientation, conductivity), the pore formation may be permanent (irreversible) or temporary (reversible) [163,172]. Depending on whether reversible or irreversible pore formation is targeted, there are approximate ranges for the treatment intensities. At low or moderate electric field intensities, when the pore size remains small relative to the total membrane surface, cells can reseal and repair their membranes after the treatment is halted, making the electroporation process reversible. However, as the electric field intensity increases and the treatment duration extends, larger pores form, leading to irreversible membrane damage and ultimately cell death [164,171,173]. For microbial cells, treatment intensities of 15–40 kV/cm are applied for microbial inactivation, whereas intensities of 0.5–1.5 kV/cm induce stress responses and reversible electroporation. Consequently, reversible electroporation is usually applied in vivo to trigger membrane permeabilization, to gain access to the cytoplasm, and to trigger stress reactions in cells [164,174,175]. Based on the studies summarized in Table 1, microbial stimulation is typically observed under sub-lethal PEF conditions employing electric field strengths below those commonly used for microbial inactivation, often in the range of approximately 0.5–5 kV/cm. However, the optimal treatment window strongly depends on pulse characteristics, treatment duration, microbial strain, and matrix composition.

##### Influence of PEF on the Cell Stress Response of Lactic Acid Bacteria

Depending on the pulse width, either smaller membrane pores can be generated by nanosecond PEF, or larger membrane pores can be induced by µPEF and mPEF. Regardless of the pulse width, PEF are fundamentally harmful to cellular homeostasis, as they disrupt membrane integrity and disturb the balance of intra- and extracellular molecules across the cell membrane [164]. Different effects of electroporation on microbial viability and metabolism have been previously documented. Currently, one of the interesting research fields of PEF application is its application to the field of fermentation. PEF treatment at moderate (sub-lethal) levels could stimulate microorganisms prior to or during fermentation, which could improve the desirable properties of the fermented products.

Table 1 gives an overview of the existing literature dealing with exertion of targeted stress by PEF on LAB. Overall, it is described that PEF treatments of LAB at sub-lethal levels lead to higher growth rates and decreased fermentation times [176,177,178,179,180,181,182,183], accelerated and/or increased lactic acid yields [180,184,185], increased proteolytic activities [177,179,184], and increased bioconversion processes [182,186,187]. Moreover, overall textural property improvements of fermented foods, such as the syneresis levels and stiffness of yogurt [177], were found. Publications concerning the increased production of EPS from microorganisms stressed by PEF remain rather scarce. However, Ohba, et al. [188,189] investigated the influence of PEF on EPS production by *Lactococcus cremoris* in a fermenter containing a 0.1% lactose solution. The PEF treatment (pulse width 1 μs; electric field strength 8 kV/cm) was applied during the mid- and late exponential growth phases, leading to an increased EPS production of around 32%.

Although the mechanism explaining why PEF stress leads to increased EPS production is unclear, there are two hypotheses that attempt to explain this effect. One hypothesis implies that the formation of pores by electroporation increases cell permeability, and thus the diffusion of ions and molecules [187,190,191,192,193], which in turn increases nutrient uptake, leading to the stimulation of EPS production [177,194]. Another hypothesis states that the electro-compressive stimulation of PEF might also induce oxidative stress responses, which activate several metabolic processes to protect the microorganisms from stress [195]. Thus, the application of PEF at sub-lethal levels to LAB is associated with their oxidative stress response [22,196]. Accordingly, high voltage discharge in cells in aqueous suspensions causes the formation of reactive oxygen species (ROS), such as H_2_O_2_, both in the medium and in the bacterial cells [197,198,199]. ROS can regulate secondary metabolism and alter enzyme activity, shifting metabolic pathways to enhance the production of fermentation metabolites [195]. The exact mechanism behind the increased production of EPS due to the application of oxidative stress in LAB is not completely understood. Nevertheless, bacteria are known to respond to increased levels of cytoplasmic H_2_O_2_ by the overproduction of EPS, along with the expression of oxidative stress response enzymes, such as NADH oxidase. Accordingly, an oxidative stress response via PEF might lead to over-expression of NADH oxidase, which is seen as key for EPS production [200,201,202].

While numerous studies report beneficial effects of sub-lethal PEF treatments on microbial growth, fermentation kinetics, and metabolite production, the magnitude and direction of these effects vary considerably depending on the microbial strain, growth phase, and processing conditions. For instance, increased growth rates and enhanced acidification have been observed in several studies [176,177,178,179,180,181,182,183,184,185], whereas other investigations report only moderate or transient effects, particularly when treatment parameters exceed optimal sub-lethal thresholds. Similarly, while enhanced proteolytic activity and bioconversion processes have been reported [177,179,182,186], these responses are highly system-specific and may depend on the membrane composition, stress tolerance, and metabolic flexibility of the respective strain. This variability highlights that PEF-induced stimulation cannot be generalized across different LAB without considering strain-specific responses. In the context of EPS production, the available literature remains limited, and reported increases are often moderate. For example, Ohba, et al. [188,189] observed a 32% increase in EPS production by *Lactococcus cremoris* under specific treatment conditions, whereas comparable studies are scarce. This indicates that, although promising, the stimulation of EPS synthesis via PEF is not yet fully understood and requires further systematic investigation. Moreover, most existing studies have been conducted in simplified media or dairy-based systems, which differ substantially from plant-based matrices in terms of composition, ionic strength, and structural complexity. As a result, the transferability of these findings to plant-based cream cheese alternatives remains uncertain. Matrix-specific factors, including protein structure, lipid distribution, and the presence of dietary fibers, may significantly influence both electroporation efficiency and microbial stress responses. Overall, while PEF represents a promising tool for modulating microbial activity and enhancing fermentation performance, its application in complex plant-based systems requires further investigation to better understand the interactions among processing parameters, microbial physiology, and matrix composition.

##### Challenges of PEF Application for Stimulation

Since PEF treatment involves passing electric currents at high voltages through a product, the conductivity is the parameter that likely poses the greatest challenge of this technology [203]. It is known that conductivity affects the efficacy of PEF treatment [167,175,204,205] and is highly dependent on the composition of the products’ ingredients [206,207]. For instance, ingredients such as salts have an increasing effect on the conductivity [208,209], whereas lipids have a decreasing effect [210]. On the one hand, products with high conductivity or bubbles can cause arcing, which will drastically increase the local temperature and cause the formation of unwanted radicals [211,212]. On the other hand, food products with low conductivity may not be as efficiently treated with PEF, requiring adjustments in treatment conditions or the use of conductive additives, which could impact the final product. Therefore, it is of great importance to investigate each matrix individually.

In addition to the general technical challenges discussed above, the application of PEF in plant-based cream cheese alternatives is further complicated by the heterogeneous and multi-phase nature of these systems. Matrix components such as proteins, lipids, salts, and dietary fibers may significantly influence electric field distribution and treatment efficiency. Electrical conductivity, which strongly depends on ionic strength and composition, represents a critical parameter for PEF processing [213,214]. While salts may increase conductivity, lipids can reduce conductivity and contribute to non-uniform electric field distribution. Furthermore, the generation of reactive oxygen species (ROS) during PEF treatment has been associated with oxidative reactions in food systems [215]. In fat-containing plant-based matrices, such effects may negatively affect flavor stability and product quality. Therefore, successful implementation of PEF requires careful optimization of treatment parameters according to matrix-specific properties. Another challenge, as with most technologies, is scalability. Large-scale systems must ensure uniform treatment and efficiency, as observed at the lab scale. This challenge also implies the complexity of PEF technology. PEF systems are complex systems, offering numerous parameter adjustments, such as varying electric field strength, frequency, number of pulses, pulse widths, and overall energy input. Moreover, maintaining consistent results can be difficult due to variations in food properties. Since foods are natural products, the standardization of ingredients is always subject to certain deviations, such as moisture content and mineral composition [203].

**Table 1:** Overview of existing literature dealing with exerting targeted stress by PEF on lactic acid bacteria *.

Reference	Microorganism	Research Objective	Medium	Time of Treatment; Bacterial Concentration During Treatment	Parameter of PEF Treatment								Stress Result/Outcome
					PEF Device	Field Strength	Number of Pulses	Duration	Frequency	Pulse Shape	Pulse Width	Energy Input	
Bisson, et al. [216]	*Leuconostoc mesenteroides* DSA_O	Investigation of the potential of moderate-intensity PEF to enhance dextran production; viability, colony morphology, EPS yield, and cell surface structure were evaluated	MRS broth	During the mid-exponential phase; 10^4^ CFU/mL	M100 ScandiNova generator (Uppsala, Sweden); parallel piped treatment chamber (16.8 mL volume capacity); 1.5 cm gap between the electrodes	1.18 ± 0.02 kV/cm	300/600/900 pulses	-	250/700 Hz	Square pulse	5 μs	322.7–980.6 kJ/kg	EPS yield increased proportionally to treatment intensity, a more than seven-fold increase compared to untreated reference; no significant reduction of viability, but smaller colonies after PEF treatment were observed on solid media, suggesting a sub-lethal physiological effect; increased EPS accumulation at cell surface observed by confocal laser scanning; NMR showed that dextran branching degree remained unchanged
Miranda-Mejía, et al. [217]	*Streptococcus thermophilus*, *Lactobacillus* *delbrueckii* subsp. *bulgaricus*	Investigation of the modulation of yogurt fermentation through PEF and influence of milk fat content; pH, soluble solids, lactose, lactic acid, and riboflavin concentrations were analyzed	Milk with different fat contents (0.5–2.8%)	Lag phase	EPULSUS^®^-LPM1A-10 system (Energy Pulse Systems, Lisbon, Portugal); batch parallel treatment chamber with a 10 cm gap and insulated with a nylon dielectric material	1 kV/cm	100/150/200 pulses	800/1200/1600 µs	10 Hz	Mono-polar pulses	8 µs	-	PEF treatment reduced the fermentation time of inoculated milk by 4.3–20.4 min; milk inoculated with PEF- treated culture showed increased lactose consumption (1.6–3.1%) and higher lactic acid production (7.2%) than control; results suggest that PEF treatments promote reversible electroporation in microbial cells, easing nutrient uptake and acidification
Miranda-Mejía, Del Campo-Barba, Arredondo-Ochoa, Tejada-Ortigoza, and La Peña [176]	*Streptococcus thermophilus*, *Lactobacillus* *delbrueckii* subsp. *bulgaricus*	Investigation of the impact of PEF processing conditions on yogurt fermentation time and quality characteristics	Partially skimmed milk	Prior to fermentation: applied to the inoculated milk before fermentation stage, having LAB at their lag phase growth	EPULSUS^®^-LPM1A-10 equipment (Energy Pulse Systems, PRT); batch	1/2/3 kV/cm	50/100/150 pulses	-	50 Hz	Mono-polar square wave pulses	4/6/8 μs	-	PEF treatment decreased fermentation time compared to control; PEF treatment leading to shortest fermentation: 8 μs mono-polar pulses, 1 kV/cm, 150 Hz during 400 μs; physicochemical and sensory characteristics of PEF-treated yogurt were similar to control
Stühmeier-Niehe, Lass, Brocksieper, Chanos, and Hertel [177]	*Lactobacillus delbrueckii* subsp. *bulgaricus* LB186, *Streptococcus thermophilus* ST504	Investigation of PEF pretreatment of a dairy starter culture on the fermentation and final product properties of set-style yogurt; effects of PEF treatment parameters (voltage, pulse number, frequency, pulse width) on pH, cell counts, proteolytic activity, texture, and degree of syneresis in yogurt were investigated	Peptone water for treatment; milk (from skim milk powder) for inoculation	Prior to inoculation	PEFPilotTM Dual (Elea GmbH, Quakenbrück, Germany); chamber: volume of approx. 70 mL, 20 cm length, 5 cm width, 1 cm depth in the center; electrodes parallel distance of 20 cm	0.2–1 kV/cm	20–80 pulses	-	1–21 Hz	Mono-polar pulses with rectangular decay	5–8 μs	-	Significant effects of pulse frequency and pulse width on yogurt stiffness; significant effect of interaction of voltage and frequency on stiffness and proteolytic activity; accelerated fermentation due to PEF treatment immediately before inoculation; increased stiffness and decreased syneresis in the final yogurt were observed
Djukić-Vuković, Meglič, Flisar, Mojović, and Miklavčič [184]	*Lacticaseibacillus rhamnosus* ATCC 7469, *Listeria innocua* ATCC 33090, *Lacticaseibacillus paracasei* NRRL B-4564, *Escherichia coli* K12 Top10 with plasmid pEGFP-N1	Investigation of inactivation kinetics and permeabilization by batch and continuous PEF treatment	MRS broth	Mid exponential phase; 5 × 10^7^ CFU/mL	Batch (IGEA s.r.l., Carpi, Italy) PEF treatment; continuous flow-through chamber with built-in electrodes (d = 2 mm, volume 0.5 mL)	0.3–25 kV/cm	100 pulses	-	1 Hz	Square waves	8 μs	Specific energy input (kJ/L): 0.72–19.00 (continuous); 9–107.6 (batch)	10% higher lactic acid production was induced at sub-lethal PEF treatment (5 kV/cm, 8 × 1 ms, 1 Hz) in *L. rhamnosus*; a release of proteins (assessed using Bradford’s assay) from both bacteria was observed
Chanos, Warncke, Ehrmann, and Hertel [185]	*Streptococcus thermophilus* DIL 5218, *Lactobacillus delbrueckii* subsp. *bulgaricus* DSMZ 20081T	Investigation of the culture’s acidification capability in reconstituted skim milk medium	Reconstituted skim milk powder	Mid exponential phase; 1.5 × 10^5^ CFU/mL	ELCRACK HPV Batch PEF equipment (Elea GmbH, Quakenbrück, Germany); chamber volume of 125 mL; 3 cm electrode gap	1/3.67 kV/cm	5/50 pulses	-	0.5 Hz/4 Hz	Exponential	-	Specific energy (kJ/kg): 1.21 × 10^−4^–9.00 × 10^−7^	Acceleration of acidification of milk; decrease of the pH, lag phase up to 12 min; the total number of pulses applied was the most influencing factor
Peng, Koubaa, Bals, and Vorobiev [178]	*Lactobacillus delbrueckii* subsp. *bulgaricus* CFL1	Investigation of the effect of PEF on growth and acidification kinetics during fermentation	MRS broth	During the fermentation process	PEF-assisted fermentation: continuous flow treatment chamber	0.060–0.428 kV/cm	1–10 × 10 pulses	-	-	Mono-polar pulses of near-rectangular shape	100 μs	-	A biphasic growth pattern was observed when applying high PEF intensities (beyond 0.285 kV/cm) and a decrease in the acidification activities; cell stress was demonstrated with accelerated growth after stopping it
Ohba, Uemura, and Nabetani [188]	*Lactococcus cremoris*	Investigation of the effect of PEF on EPS biosynthesis in a fermenter	Oxoid M17 broth supplemented with 0.1% lactose	During the mid to late exponential growth phase	Flow-cell; installed via a by-pass line connected to the fermenter	8 kV/cm	150–400 pulses	-	-	-	1 μs	-	Increased EPS production by around 32%
Najim and Aryana [179]	*Lactobacillus* *acidophilus* LA-K *Lactobacillus* *delbrueckii* subsp. *bulgaricus* LB-12	Investigation of the influence of mild PEF conditions on acid tolerance, growth, and protease activity	Peptone water	-	Continuous PEF system (OSU-4M; The Ohio State University, Columbus, OH, USA); flow rate of 60 mL/min	1 kV/cm	-	-	-	Positive square unipolar pulse	3 μs	-	PEF allowed the bacteria to reach the logarithmic phase of growth an hour before the control; improved acid tolerance, exponential growth, and protease activity of both *L. acidophilus* LA-K and *L. bulgaricus* LB-12 compared with the control
Seratlić, Bugarski, Nedović, Radulović, Wadsö, Dejmek, and Galindo [181]	*Lactiplantibacillus plantarum* 564	Investigation of the effect of PEF with amplitudes below 14 kV/cm and the applied energy up to 12.2 J/cm^3^ on the growth of *L. plantarum* 564 cells	Sterilized distilled water; at a ratio of 1:10; conductivity of approx. 1.3 mS/cm	During the early and mid-exponential growth phases	CEPT pulse generator (Arc Aroma Pure, Lund, Sweden)	4.5/9.1/13.6 kV/cm	10	-	100 Hz	Mono-polar square wave pulses	5 μs	1.33/5.5/12.2 J/cm^3^	Higher growth rate of PEF-treated cells during the early and mid-log phases, especially bacterial samples treated with lower field intensities (1.3–5.5 J/cm^3^); doubling time was reduced after treatment at 5.5 J/cm^3^; better acidification ability of electroporated cells
Seratlić, Bugarski, and Zorica Radulovi [180]	*Lactiplantibacillus plantarum* 564	The behavior of the surviving population of *L. plantarum* 564 growing in MRS broth after PEF treatments was investigated (by isothermal calorimetry, optical density, and plate counts)	Sterilized distilled water; at a ratio of 1:10; conductivity of approx. 1.3 mS/cm	During the early and mid-exponential growth phases; 8.6 × 10^6^ CFU/mL	CEPT pulse generator (Arc Aroma Pure, Lund, Sweden)	22.9/31.6 kV/cm	10/100	-	100 Hz	Mono-polar squared pulses	5 μs	34.6/65.8/658.1 J/cm^3^	The surviving bacteria resumed growth after a treatment-dependent delay; untreated and treated cultures had similar growth rates; PEF-treated culture showed a higher growth rate during the late-growth phase; growth rate increased with the intensity of applied electric field
Yeo and Liong [182]	*Bifidobacterium* sp. FTDC 8943, *B. longum* FTDC8643, *Lacticaseibacillus casei* FTDC2113, *Lacticaseibacillus casei* BT 1268	Investigation of the effects of electroporation on bacterial growth, membrane properties, and bioconversion of isoflavones	Mannitol-soy milk	-	Gene Pulser II with integrated Pulse Controller Plus (Bio-Rad Laboratories, Hercules, CA, USA); 0.2 cm electrode gap size	2.5; 5; 7.5 kV/cm	-	3; 3.5; 4 ms	-	-		-	Reversible decrease in viability after electroporation; increased growth rate; increased bioconversion of glucosides to bioactive aglycones in mannitol-soy milk after electroporation
Ewe, Wan-Abdullah, Alias, and Liong [186,187]	*Limosilactobacillus fermentum* BT 8219	Investigation of the effects of electroporation on growth, isoflavone bioconversion activities, and probiotic properties	Biotin- supplemented soy milk	Prior to inoculation and fermentation in biotin-soy milk	MicroPulser (Bio-Rad Laboratories, Hercules, CA, USA); 0.2 cm electrode gap	2.5; 5.0; 7.5 kV/cm	-	3; 3.5; 4 ms	-	-	-	-	Electroporation caused cell death, followed by increased growth during fermentation compared to control; the bioconversion process was increased at higher-intensity electric PEF (7.5 kV/cm, 3.5 ms) due to increased intra- and extracellular ß-glucosidase activities; structural changes within the cellular membrane of lactobacilli were triggered by electroporation that caused lipid peroxidation and alteration of membrane fluidity
Lye, Karim, Rusul, and Liong [183]	*Lactobacillus acidophilus* BT 1088, *L. acidophilus* FTCC 0291, *L. bulgaricus* FTCC 0411, *L. bulgaricus* FTDC 1311, and *Lacticaseibacillus casei* BT 1268	Investigation of electroporation on the membrane properties of lactobacilli and their capacity for cholesterol removal in vitro	Sucrose solution (1 mM)	Prior to fermentation	MicroPulser, (Bio-Rad Laboratories, Hercules, CA, USA)	2.5; 5; 7.5 kV/cm	-	3; 3.5; 4 ms	-	-	-	-	Increased growth of lactobacilli treated at 7.5 kV/cm for 4 ms (by 0.89 to 1.96 log10 CFU/mL); reversible pore formation was observed; electroporation induced lipid peroxidation in the cellular membrane and increased membrane permeability and cholesterol uptake from the medium

* It should be noted that a considerable proportion of the studies summarized in Table 1 were conducted in dairy or model systems rather than in plant-based matrices. This reflects the current state of research, as studies specifically investigating PEF-assisted fermentation in plant-based dairy analogs remain limited. Nevertheless, these studies provide valuable mechanistic insights into microbial stress responses, metabolic modulation, and fermentation dynamics under ultrasonic treatment. Such knowledge is considered transferable to plant-based systems to a certain extent, although differences in matrix composition, such as protein structure, fat distribution, and the presence of dietary fibers, may significantly influence the observed effects. Therefore, further research is required to validate these findings in complex plant-based food systems.

#### 3.2.2. Ultrasound (US)—Mechanism of the Technology

Ultrasound (US) is defined as the energy generated by sound waves at frequencies above the threshold of human hearing, ranging from 20 kHz up to the MHz range. The spectrum of US can be divided into high-power and low-power US, which depends on the frequency, and therefore the resulting energy input. Low-power US refers to higher frequencies starting from around 1 MHz resulting in a lower energy input of below 1 W/cm^2^. These low energy inputs enable the use of US for non-invasive medical and industrial imaging applications. In contrast, high-power US refers to lower frequencies ranging from 20 kHz to 1 MHz, resulting in high power intensities above 5 W/cm^2^ [24,218,219,220] and can cause material alterations [24,221]. Depending on the frequency used and the sound wave amplitude applied, a number of physical, chemical, and biochemical effects can be observed that enable a variety of applications [218,219]. A fundamental mechanism of US is US-induced cavitation, which occurs when sound is transmitted in liquids. An ultrasound wave propagating through a liquid consists of alternating acoustic positive and negative pressure phases. These cyclic pressure variations may induce cavitation when the negative pressure amplitude drops below the vapor pressure of the medium, resulting in the formation of vapor-filled cavitation bubbles. These cavities grow over time and collapse when they reach a certain size. This collapse generates energy for mechanical and chemical effects, which include shock waves (~1000 bar), temperature hotspots (~4000 K), shear forces, local turbulences, the formation of radicals, microjets (with velocities ~400 km/h), and shifts in pH values [24,218,222,223].

US processing is distinguished by two types of cavitation, stable and transient cavitation, which behave differently and have distinct effects on food systems [224]. Stable cavitation occurs when gas bubbles oscillate over multiple US cycles without collapsing. These oscillations generate microstreaming and shear forces, which enhance mixing and improve mass transfer. In food processing, this effect can be beneficial for emulsification, homogenization, and improving the permeability of cell membranes, such as in sonoporation, where US helps extract bioactive compounds from plant or microbial cells [225,226,227]. In contrast, transient cavitation involves bubbles growing rapidly until they become unstable and collapse violently. This collapse generates extreme pressures and high temperatures, leading to the formation of shock waves and microjets. These intense forces can break down particles, disrupt cell structures, and enhance extraction efficiency [227]. In food applications, transient cavitation is particularly useful in processes such as particle size reduction, degassing, and microbial inactivation, where strong mechanical effects improve product quality and safety [228]. In summary, stable cavitation is primarily used for controlled, gentle modifications, whereas transient cavitation provides powerful mechanical forces for breaking down structures and enhancing food processing efficiency.

Moreover, the effects of cavitation vary depending on the homogeneity of the sample. At the interface of a two-phase system with a heterogenous liquid phase, cavitation bubbles are strongly deformed, and microjets arise on the interface. Finely dispersed droplets form to create emulsions. US-generated emulsions show a smaller droplet size compared to conventionally generated emulsions and are more stable. In particle suspensions, the effects of cavitation are strong enough to disrupt the particles, cause depolymerization, and thus increase their specific surface area. With fine particles, cavitation leads to the collision of these fine particles, followed by an abrasion of the particles’ surface, changing their physicochemical properties [221,223,228]. High-power US can be applied in various food treatments, as the energy input impacts the physical and (bio)chemical properties of foods. High-power US might be used in food processing to modify the textural characteristics of fat, perform emulsification, defoaming, mixing, and homogenization, modify the functionality of proteins and fibers, inactivate/accelerate enzymatic activity, and inactivate microbes [221,229,230]. In addition to microbial inactivation, US technology can also be used for the targeted stressing of microorganisms, such as LAB, as explained in the following section. Similar to PEF, beneficial stress responses induced by ultrasound are generally associated with moderate treatment conditions, where cavitation effects are sufficient to stimulate microbial activity without causing extensive cell damage. However, reported frequencies, power intensities, and treatment times vary considerably among studies, highlighting the need for strain- and matrix-specific optimization.

##### Influence of US on the Cell Stress Response of Lactic Acid Bacteria

Different effects of US on microbial viability and metabolism have been previously documented. For stressing, moderate energy inputs at sub-lethal levels are needed to stimulate microorganisms prior to or during fermentation, which could improve the desirable properties of the fermented products. It must be emphasized that the effects of US on LAB were found to be strain-dependent [231] and dependent on the applied ultrasonic power and treatment time [232,233]. Table 2 gives an overview of the existing literature dealing with exerting targeted stress by US on LAB. Overall, it is described that US treatments of LAB increased the fermentation efficiency. A decrease in fermentation time [232,234] and the promotion of the growth of different LAB strains has been reported [231,235,236]. Moreover, increases in biomass production and substrate consumption were observed [236], as well as alterations of bioactive properties during fermentation. These bioactive properties include increased lactose hydrolysis and trans-galactosylation reactions [237], α-amylase inhibition, α-glucosidase inhibition, antioxidant activity, anticancer activity [231], increased β-glucosidase activity, and increased bioconversion of isoflavone glucosides to aglycones [186,187,238,239]. Dahroud, Mokarram, Khiabani, Hamishehkar, Bialvaei, Yousefi, and Kafil [236] and Nguyen, Lee, and Zhou [237] observed increases in lactic acid contents, whereas no significant differences in lactic acid content were observed by Ojha, Kerry, Alvarez, Des, and Tiwari [232] or Düven, Kumcuoğlu, and Kışla [234].

In general, increased EPS production and enhanced EPS biosynthesis were observed as effects of high-intensity US pretreatment of LAB [231,233,235,240]. Additionally, US-treated yogurts showed increased gel stability, indicating EPS as a tool to reduce syneresis [241]. The concrete mechanism explaining the stress response of LAB to US treatment remains unclear. However, there are existing hypotheses that attempt to explain the above-mentioned effects. It was observed that US treatment leads to increased cell membrane permeability [235,236,242] and cell membrane porosity [186,243,244]. More specifically, it was shown that US treatment affected the fatty acids chain of the cellular membrane lipid bilayer, shown by increased lipid peroxidation, leading to increased membrane fluidity and membrane permeability. These permeabilized cellular membranes were seen to improve mass transfer, which might facilitate nutrient internalization and thus lead to growth enhancement due to higher production rates [186,187,233,238,239,245]. Another hypothesis states that the cavitation-based rupture of cell membranes during US treatment might release growth-promoting factors, such as amino acids, nucleotides, enzymes, and vitamins, that could stimulate the growth of undamaged bacterial cells [233,246,247,248]. Alternatively, due to the quick changes in vapor pressure during US treatment, the induction of stress via pressure fluctuations on cells is hypothesized, which might promote cell growth and proliferation, as well as changes in metabolic pathways [233,239].

Although numerous studies report beneficial effects of ultrasound on microbial growth, fermentation kinetics, and metabolic activity, the observed responses vary substantially depending on the applied treatment conditions, microbial strain, and matrix composition. Moderate ultrasound intensities have frequently been associated with enhanced microbial activity, accelerated fermentation, and improved mass transfer, whereas excessive treatment intensity or prolonged exposure may lead to irreversible cell damage and reduced viability [221,229,230,231,232,233]. Similarly, improvements in bioconversion processes and enzymatic activities have been reported in several studies [234,235,236,238,249], yet these effects appear to be highly strain- and process-dependent. Such variability likely results from differences in cell membrane composition, cavitation sensitivity, and stress adaptation mechanisms among LAB. Furthermore, while US-induced cavitation may enhance nutrient transport and microbial metabolism, it may also generate localized high temperatures, pressure gradients, and reactive radicals, which can negatively affect microbial stability and food quality under inappropriate processing conditions [215,219,220]. Therefore, careful optimization of treatment parameters is essential to balance stimulatory and detrimental effects. It should also be noted that a considerable proportion of the available studies have been conducted in dairy or simplified model systems. Studies specifically investigating US-assisted fermentation in plant-based dairy analogs remain scarce. Consequently, the transferability of these findings to plant-based cream cheese alternatives remains uncertain, as matrix-specific factors such as protein structure, fat distribution, viscosity, and the presence of dietary fibers may significantly influence cavitation behavior, microbial responses, and final product characteristics. Overall, while US represents a promising approach for modulating microbial activity and fermentation processes, further research is needed to better understand its effects in complex plant-based matrices and to evaluate its technological feasibility at an industrial scale.

**Table 2:** Overview of the existing literature dealing with exerting targeted stress by ultrasound (US) on lactic acid bacteria (LAB) *.

Reference	Microorganism	Research Objective	Medium	Time of Treatment; Bacterial Concentration During Treatment	Parameter of US Treatment						Stress Result/Outcome
					US Device	Volume	Frequency	Duration	Power/Intensity	Other Operating Details	
Costa, et al. [250]	*Lactococcus lactis* AQLM2 and *Lacticaseibacillus rhamnosus* 6QLR4	Investigation of the technological potential of US application during milk fermentation with LAB; evaluation of its effects on the development of value-added ricotta cheese by analyzing volatile compounds; physicochemical, microbiological (viable cell count), and sensory attributes of the final product were analyzed	Fermented milk (reconstituted skim milk powder (Elegê^®^, Lactalis do Brasil, Teutônia, Brazil))	In between fermentation	Ultrasonic bath (USC 1450, Unique, São Paulo, Brazil)	100 mL	25 kHz	0/5/10/15 min	150 W	-	No significant differences (*p* ≥ 0.05) observed among viable cell counts of fermented milk subjected to different US treatment times; US treatment (10 min) enhanced the production of volatile compounds, reflecting increased metabolic activity of the LAB
Gholamhosseinpour, et al. [251]	*Lactobacillus helveticus* PTCC 1332	Investigation of the impacts of US on fermentation dynamics and bioactive properties in fermented milk; cell viability, pH, DPPH radical scavenging activity, and inhibition of ascorbate autoxidation enzyme activities were analyzed	UHT milk	Prior to fermentation; bacteria sonicated and added to milk/milk without bacteria sonicated and then bacteria added to milk/bacteria and milk sonicated separately before combining/bacteria added to milk and then sonicated	Ultrasonic bath (Elmasonic S 300H; 37 KHz, 300 W; Germany)	50 mL	-	150 s	-	US treatment at 30 °C	US significantly increased microbial viability and accelerated acidification; a synergistic effect was observed when US was applied to milk and led to the most pronounced improvements in bacterial growth, acidification, antioxidant capacity, and enzyme-inhibitory activities
Encinas-Vazquez, Carrillo-Pérez, Mártin-García, Del-Toro-Sánchez, Márquez-Ríos, Bastarrachea, and Rodríguez-Figueroa [240]	Kefir grains	Investigation of the effect of high-intensity US on kefir grains biomass increase and metabolites in cheese whey kefir; investigated parameters: total EPS production, kefir grain biomass increase, titratable acidity, pH, and soluble solids	Cheese whey kefir	Prior to inoculation and fermentation	Ultrasonic horn (22 mm diameter and 100 mm length, S24d22D, Hielscher Ultrasonic, Germany); 400 W US equipment (UP400St, Hielscher Ultrasonic, Germany)	600 mL	23.0 ± 0.9 kHz	30/180 s	9.0 ± 2.7 W/cm^2^; 18 ± 3.0 W/cm^2^ (70% amplitude)	Cooling during treatment; tip of the probe 3 cm deep in the sample solution	High-intensity US pretreatment enhanced the biosynthesis of kefir beverage total EPS concentration and kefir grain biomass; cheese whey kefir pretreated with 18.0 ± 3.0 W/cm^2^, 180 s, fermented for 16 h showed significantly higher total EPS concentration than the control
Bagher Hashemi, et al. [252]	*Lactiplantibacillus plantarum* LS5 and LU5	Investigation of the effects of US pretreatment on EPS production, EPS quality, and bioactive properties of extracted EPS (antibiofilm, α-amylase inhibition, cholesterol-lowering, and antioxidant activities)	MRS broth supplemented with sucrose (20 g/L)	Inoculated MRS broth was treated prior to fermentation	US probe (Hielscher ultrasonic device; UP100H; Germany; 100 W, 30 kHz)	-	30 kHz	15 min	Max. 100 W (0/25/50/75% amplitude)	Cooling during treatment	LS5-EPS showed higher bioactivity than LU5-EPS; US pretreatment at 50% amplitude was most efficient for increasing EPS production by LS5
Düven, Kumcuoğlu, and Kışla [234]	Commercial kefir starter culture powder	Investigation of microbial activity enhancement effects of US waves on kefir fermentation; parameters: microbiological analysis, pH, titratable acidity (lactic acid %), produced EPS	Full-fat UHT milk	-	Hielscher UP400S, 400 W, 22 mm probe (New Jersey, USA)	400 mL	24 kHz	5 min	400 W (30% amplitude)	-	Decrease in fermentation time by 1 h; no sign. increase in EPS and lactic acid production rates
Hashemi and Gholamhosseinpour [231]	*Lactiplantibacillus plantarum* LP3 and LU5 strains	Investigation of the effects of continuous US treatment and fermentation on the growth of *Lactobacillus* strains and the bioactive properties (peptide content, α-amylase and α-glucosidase inhibition, anticancer and antioxidant activities, and EPS content) of goat milk	Goat milk	Sonication was performed after inoculation	Hielscher ultrasonic device (UP100H; Germany)	100 mL	30 kHz	15 min	100 W (0/30/60/90% amplitude)	Continuous sonication mode; cooling during treatment	60% amplitude showed promotion of the growth of *Lactiplantibacillus* strains and enhancement of bioactive properties compared with control during fermentation; EPS content, α-amylase inhibition, α-glucosidase inhibition, antioxidant activity, and anticancer activity were increased; increase in assayed characteristics was found to be strain-dependent; 90% amplitude showed negative effects on the mentioned parameters
Khadem, Tirtouil, Drabo, and Boubakeur [235]	*Streptococcus thermophilus* CNRZ 447	Investigation of the effects of US conditioning on the metabolism and extracellular matrix production of *Streptococcus thermophilus;* investigated parameters: growth improvement, adhesion ability, biofilm formation, and EPS production of the bacterial strain	M17 broth	Bacterial suspensions (in glass test tubes) were treated after inoculation; 10^7^ CFU/mL	Sonorex ultrasonic bath (35 kHz, 240/60 W peak/nominal powers, 1.8 L capacity) (SONOREX TK 52)	10 mL	35 kHz	5/10/15/20/30/45/65 min	-	At 25 °C	Improvement of growth, adhesion, membrane permeability, biofilm formation, and EPS production ability; optimal effect was obtained after 30 min treatment: increased EPS content was observed
Liu, Yang, and Fang [239]	*Lactobacillus acidophilus* BCRC 10695, *L. bulgaricus* BCRC 10696, *L. casei* BCRC 14080, *Streptococcus thermophilus* BCRC 13680, *S. thermophilus* ATCC BAA-250	Development of methodology for strategic ultrasound treatment of lactic acid bacteria to induce a stress response for the enhancement of β-glucosidase activity	Treatment carried out in MRS broth; fermentation carried out in soy milk	Treatment during stationary phase (24 h), prior to inoculation and fermentation	Sonicator Q700, QSONICA, USA; max. 700 W; tip of probe: 1.27 cm × 12.7 cm	-	20 kHz	-	40 W (20% amplitude)/ 50 W (30% amplitude)/ 60 W (40% amplitude)/ 80 W (60% amplitude)	Tip of the probe 1 cm deep in the sample solution	US treatment of *L. acidophilus* BCRC 10695 showed increased β-glucosidase activity; *L. acidophilus* BCRC 10695 showed the best ability to release β-glucosidase (20 kHz, amplitude at 20%, 2 min); increased bioconversion of isoflavones glucosides to aglycones
Körzendörfer, Nöbel, and Hinrichs [241]	*Lactobacillus* *delbrueckii* subsp. *bulgaricus* and *Streptococcus thermophilus* strains: YC-471 (low EPS) or YF-L 901 (high EPS) (Chr. Hansen)	Investigation of the impact of sonication during fermentation with starter cultures differing in EPS synthesis on the physical properties of set and stirred yogurt (syneresis, firmness, particle size, and rheology)	Skim milk	Skim milk fermented with starter cultures; sonicated at pH 5.2; 42 °C	300 W, V= 19 L; RK 1028/H; Bandelin electronic GmbH & Co. KG, Berlin, Germany	100 mL	35 kHz	5 min	-	-	Sonicated set gels showed higher syneresis levels than control; sonication significantly increased particle numbers, but the effect was less pronounced when high EPS culture was used, indicating EPS as a tool to reduce syneresis and particle formation due to vibrations
Dahroud, Mokarram, Khiabani, Hamishehkar, Bialvaei, Yousefi, and Kafil [236]	*Lacticaseibacillus casei* subsp. *casei* ATTC 39392	Investigating the application of low intensity US technology to improve the metabolic activity of l-lactic acid production by *L. casei* in different media; parameters of fermentation yield: lactic acid, biomass production, and substrate (protein) consumption	MRS broth with the addition of different peptone levels: 2/6/10 g/L	Sonication 2 h after inoculation (when cells entered the logarithmic phase); sonication was applied during the log phase every 2 h	UP200H model; 0.5 < cycle < 1, 20% < amplitude < 100%, Hielscher, Germany	50 mL plastic falcon	-	15/30/45 s	20/40/60% amplitude	Tip of the probe 1 cm deep in the sample solution; cooling during treatment	US treatment increased the fermentation efficiency: lactic acid, biomass production, and substrate consumption significantly increased (≈ 25%); optimum conditions for biomass production: amplitude of 60%, 15 s exposure time, 10 g/L peptone addition; optimum conditions for lactic acid production and substrate consumption: 40%, 30 s, and 6 g/L peptone addition; flow cytometry analysis showed that sonication led to increasing cell membrane permeability
Ojha, Kerry, Alvarez, Des, and Tiwari [232]	*Latilactobacillus sakei* DSM 15831	Investigation of the efficacy of high intensity ultrasound on the fermentation profile of *L. sakei* in a meat model system	MRS broth with meat extract added	Inoculated solution (1 × 10^6^ CFU/mL) sonicated prior to fermentation	Ultrasonic probe (XL2020 Heat Systems, Misonix Inc., Farmingdale, NY, USA; 550W); 13 mm diameter ultrasonic probe	100 mL	20 kHz	0–9 min	0–68.5 W	-	Stimulation and retardation of *L. sakei* was shown, depending on the ultrasonic power and treatment time; a higher specific growth rate and a shorter lag phase were observed at low power (2.99 W for 5 min) compared to control; a decrease in the specific growth rate with an increase in the lag phase was observed with an increase in ultrasonic power level; no significant difference was observed for lactic acid content
Ewe, Wan Abdullah, Bhat, Karim, and Liong [249]	*Lactobacillus acidophilus* BT 1088, *Limosilactobacillus fermentum* BT 8219, *L. acidophilus* FTDC 8633, *L. gasseri* FTDC 8131	Investigating the effect of US treatment to enhance the growth of lactobacilli and their isoflavone bioconversion activities in biotin-supplemented soy milk	Biotin-supplemented soy milk	Inoculated phosphate-buffered saline (pH 7.4) US-treated prior to inoculation and fermentation in biotin-supplemented soy milk	Ultrasonic Processor (30 kHz; power output 100 W; LABSONIC^®^ M, Sartorius Stedim Biotech), with 80 mm long, 3 mm diameter titanium sonotrode	10 mL	30 kHz	60/120/180 s	100 W (20/60/100% amplitude)	Tip of the probe 1 cm deep in the sample solution	US treatment affected the fatty acid chains of the cellular membrane lipid bilayer, shown by an increased lipid peroxidation, leading to increased membrane fluidity and membrane permeability; those permeabilized cellular membranes facilitated nutrient internalization, leading to growth enhancement; higher amplitudes and longer durations of the treatment promoted growth of lactobacilli; US treatment increased intracellular and extracellular ß-glucosidase activities, leading to increased bioconversion of isoflavones in soy milk
Ewe, Wan-Abdullah, Alias, and Liong [238]	*Limosilactobacillus fermentum* BT 8633	Evaluation of the effects of US on *L. fermentum* BT 8633, based on growth and isoflavone bioconversion activities in biotin-supplemented soy milk	Biotin-supplemented soy milk	Inoculated phosphate-buffered saline (pH 7.4) US-treated prior to inoculation and fermentation in biotin-supplemented soy milk	Ultrasonic Processor (30 kHz; power output 100 W; LABSONIC^®^ M, Sartorius Stedim Biotech), with 80 mm long, 3 mm diameter titanium sonotrode	10 mL	30 kHz	2 min	60 W (60% amplitude)	Tip of the probe 1 cm deep in the sample solution	US treatment increased the growth of *L. fermentum* in biotin-supplemented soy milk after fermentation; enhanced intracellular and extracellular b-glucosidase activity of cells was observed, which increased the bioconversion of glucosides to aglycones in biotin-supplemented soy milk; the effect of US was transient and not inherited to following generations of cells
Nguyen, Lee, and Zhou [237]	*Bifidobacterium breve* ATCC 15700, *Bifidobacterium longum* subsp. *infantis*, *Bifidobacterium animalis* subsp. *lactis B. lactis* (BB-12), *B. longum* (BB-46)	Investigation of the effect of high intensity US on carbohydrate metabolism in milk fermentation	Reconstituted skimmed milk; inoculated with bacteria in the early stationary phase	At the beginning of fermentation, inoculated milk samples were sonicated	UIP 1000 US system, Sonotrode BS2d34 (Hielscher Ultrasonics GmbH, Teltow, Germany)	100 mL	20 kHz	7; 15; 30 min	~100 W (30% amplitude)	Cooling during treatment	After US treatment: lactose hydrolysis and the trans-galactosylation reaction in all fermented milk were accelerated; increased lactose consumption and changes in acid profiles of the strains were observed

* It should be noted that a considerable proportion of the studies summarized in Table 2 were conducted in dairy or model systems rather than in plant-based matrices. This reflects the current state of research, as studies specifically investigating ultrasound-assisted fermentation in plant-based dairy analogs remain limited. Nevertheless, these studies provide valuable mechanistic insights into microbial stress responses, metabolic modulation, and fermentation dynamics under ultrasonic treatment conditions. Such knowledge is considered transferable to plant-based systems to a certain extent, although differences in matrix composition, such as protein structure, fat distribution, and the presence of dietary fibers, may significantly influence the observed effects. Therefore, further research is required to validate these findings in complex plant-based food systems.

##### Challenges of US Application for Stimulation

There are different challenges associated with US applications when it comes to the stimulation of microorganisms. Firstly, for the US-induced cavitational effects, liquid media are needed for the transmission of sound waves. Moreover, the viscosity of the treated medium plays an important role in the efficiency of US treatment. Highly viscous samples, such as samples including high amounts of ingredients with high water-binding capacities, are at risk of not being treated homogeneously. This might lead to temperature hotspots and overtreated parts, as well as less intensely treated parts, within a single sample [229,236]. Furthermore, due to the emulsifying and particle-rupturing effects of US treatments, changes in texture, mouthfeel, and taste might result [219,220]. Since there is a fine line between the stimulation and inactivation of microorganisms as an effect of US treatment [253], the parameter setups for stimulating microorganisms are highly dependent on the strain [231] and dependent on the applied ultrasonic power and treatment time [232,233]. In addition to the general technical challenges discussed above, the application of US in plant-based cream cheese alternatives may be further complicated by the heterogeneous matrix composition, which can influence acoustic wave propagation and cavitation behavior. Components such as fat droplets, proteins, and dietary fibers can attenuate or scatter ultrasonic waves, thereby affecting cavitation intensity and process efficiency [254,255]. In addition, ultrasonic cavitation generates localized high temperatures and pressure gradients, which may promote lipid oxidation and induce structural protein modifications [219,256]. These effects are particularly relevant in fat-rich plant-based matrices and may negatively impact sensory quality and shelf life. Consequently, US processing requires careful adjustment of treatment conditions to balance beneficial microbial stimulation with product quality preservation.

Furthermore, the scalability of US application represents a challenge. Large-scale systems must ensure uniform treatment and efficiency, as observed at the lab scale. Depending on the design and dimensions of the sonotrode, the applied amplitudes and power inputs, as well as the volume and different overall energy inputs, may lead to different effects on the media [24,253].

## 4. Fermentation with EPS-Producing Starter Cultures: (Mechanistic) Insights in Acid-Gelled Dairy and Plant-Based Dairy Analog Matrices

Provided that the selected ingredients and processing conditions enable sufficient in situ EPS production to induce structural and, consequently, textural modifications, EPS can enhance product stability and organoleptic properties, as discussed above (Section 3). The extent to which in situ-produced EPS affect the properties of dairy products and their analogs further depends on the type, molecular structure, and functionality of the EPS formed, as well as on the composition of the target food matrix [257]. These factors influence interactions such as protein–polysaccharide associations, which contribute to structure formation in fermented dairy products and plant-based alternatives. As explained in Section 2, matrix formation in dairy and plant-based systems is governed by distinct physicochemical mechanisms arising from differences in protein structure and functionality. The following section therefore focuses primarily on fermentation and, in particular, on the role of in situ-produced EPS in acid-gelled dairy matrices. The insights provided are further discussed in the context of fermented dairy analogs, including yogurt and cream cheese alternatives.

The impact of in situ EPS formation on the characteristics of dairy products has been extensively investigated. Madhubasani, et al. [258] examined the effects of EPS-producing starter cultures on the microbiological, physicochemical, and sensory properties of probiotic goat’s milk set yogurt. Their findings indicated that in situ EPS production effectively mitigated spontaneous syneresis, defined as whey separation resulting from gel shrinkage during storage [259], a phenomenon often associated with decreased consumer acceptance [260]. Comparable results have been reported for cow’s milk set yogurt [17,261]. Furthermore, Madhubasani, Prasanna, Chandrasekara, Gunasekara, Senadeera, Chandramali, and Vidanarachchi [258] observed that the total EPS concentration appeared to influence the apparent viscosity of goat’s milk set yogurt during storage and concluded that further research is required to examine the mechanisms by which protein–EPS interactions affect the rheological properties of this product. The results are in agreement with Liu, et al. [262], who investigated the influence of EPS produced in goat’s milk by strains of *Limosilactobacillus fermentum*, *Lactiplantibacillus plantarum*, *Lactobacillus helveticus*, and *Pediococcus acidilactici*. The authors showed that these strains can enhance the texture of fermented goat’s milk and related products. Moreover, the EPS produced by the different strains varied in both monosaccharide composition and molecular weights (ranging from 6.42 × 10^3^ to 2.41 × 10^4^ Da), which likely explains the observed differences in textural attributes among samples. The influence of in situ EPS production by LAB on the textural properties of cow’s milk yogurt has also been studied in a more mechanistic context, considering the interactions between EPS and other components of the yogurt matrix. Amani, et al. [263] reported that yogurt manufactured with EPS-producing starter cultures exhibited improved textural properties compared to yogurts prepared with non-EPS-producing cultures. This effect was attributed to interactions between in situ-formed EPS and casein micelles, as well as whey proteins, which can promote network formation and modify gel firmness. EPS localization within the protein matrix and specific EPS–protein interactions appeared to play a key role in texture development, rather than EPS concentration alone. Similarly, Li, et al. [264] observed a positive correlation between EPS production and yogurt viscosity. Specifically, the secretion of the polymer EPS-S11 by *Lacticaseibacillus paracasei* H9 was associated with increased viscosity. Furthermore, experiments conducted in sodium caseinate model systems demonstrated that increasing concentrations of EPS-S11 led to higher zeta potential, reduced particle size, and decreased turbidity, supporting the conclusion that EPS-S11 interacts with sodium caseinate and contributes to structural stabilization. In addition, EPS have been shown to enhance the water-holding capacity of fermented cow’s milk [265], and fermented goat’s milk [262]. At pH values below the isoelectric point, proteins carry a positive charge and can interact with negatively charged EPS through electrostatic attraction, thereby promoting texture formation. The textural properties of yogurt, including plant-based alternatives (as subsequently highlighted), are hence influenced by the charge and structural characteristics of the EPS formed, including factors such as backbone stiffness, degree of branching, and molecular weight [141,266]. These attributes affect the interactions between EPS and the yogurt matrix, as also discussed in a study on EPS-containing yogurts by Tiwari, Kavitake, Devi, and Halady Shetty [17]. Protein–EPS interactions may also contribute to delayed gel formation, as reported by Gentès, St-Gelais, and Turgeon [141], who investigated various *Streptococcus thermophilus* and *Lactobacillus delbrueckii* subsp. *bulgaricus* strains producing EPS with differing charge characteristics (neutral or anionic) and degrees of branching. The authors observed that early production of an anionic, linear EPS by *S. thermophilus* 2104 was associated with a delayed start of gelation, which occurred at lower pH values. This phenomenon was attributed to electrostatic repulsion between negatively charged casein micelles (above their isoelectric point at approximately pH 4.6) and the anionic EPS, which interfered with protein aggregation. Although gelation was postponed, gels eventually formed and exhibited altered rheological properties, including higher viscosity and reduced syneresis compared to products fermented with control strains. Brüls, et al. [267] performed a microstructural analysis of yogurt gels produced with EPS-forming strains (neutral and anionic EPS), combining advanced microscopy and rheological testing. Neutral EPS were embedded within the casein network, acting as fillers that promoted network restructuring by inducing thicker protein domains and an abundance of small pores, rather than primarily reinforcing junction zones. Their interactions with the matrix led to enhanced gel strength and reduced syneresis through stabilization of pore architecture and the water-binding capacity of the EPS. Studies have also highlighted the great potential of EPS-forming strains to improve the creaminess, mouthfeel, and viscosity of fat-reduced dairy products including reduced-fat stirred yogurt [268] and low-fat cheese products including low-fat cheddar cheese [269], which were manufactured with an adjunct EPS-producing culture (*Lactiplantibacillus plantarum* JLK0142). Products had significantly higher moisture contents and lower hardness compared to a control cheese produced with a non-EPS-producing culture (*L. lactis* ST25). The results could be explained by the water-binding capacity of EPS, and higher moisture retention was linked to a softer texture of the end products. These results were in accordance with other studies conducted on low-fat cheeses [270,271]. Surber, et al. [272] examined the influence of different EPS-forming *L. lactis* strains and homogenization pressures (0.05, 15, and 30 MPa) on the texture and syneresis attributes of cream cheese. At homogenization pressures of 15–30 MPa, non-ropy EPS were found to enhance both serum retention and firmness, while ropy and capsular EPS were most effective at lower pressures (0.05–15 MPa), highlighting the importance of processing parameters next to strain selection.

In the fermented plant-based dairy alternatives sector, research in recent years has mainly focused on yogurt analogs, as reflected by the growing number of studies addressing plant-based yogurt alternatives and the impact of fermentation on textural properties and overall product quality, as reviewed by Montemurro, et al. [273]. Two very recent studies from Libberecht, et al. [274] and Lu, et al. [275] examined the in situ production of dextran and its impact on yogurt analog quality, the latter being based on a rice flour matrix. Libberecht, Ristevska, Gibis, and Loeffler [274] examined the potential of a HoPS-producing *Latilactobacillus sakei* strain in pea protein-enriched coconut yogurt analogs, formulated with or without starch addition, with a particular focus on texture development and product syneresis. The authors reported that in situ-produced dextran positively affected gel network formation, structural stability with respect to syneresis, and textural properties compared with products fermented using a non-EPS-producing strain, although starch partly dominated the EPS-related effects on texture and water-holding capacity. Both in situ EPS production and intrinsic polymer characteristics determine whether EPS become incorporated into the forming gel network and/or remain in the continuous phase, where they can e.g., take up a pore-filler-like role and contribute mainly to thickening, with different effects of dextran being reported by several other authors [275,276,277]. Consequently, textural modifications observed in yogurt analogs containing thickening agents such as starch may be additive or synergistic, depending on the dominant EPS mechanism within the matrix. Future research should therefore place greater emphasis on microstructural characterization, for example by cryo-SEM, to better understand these structure–function relationships [278], which are also relevant for the development of other dairy analogs. To date, research on plant-based (cream) cheese alternatives has primarily focused on the influence of protein source [279] and on the addition of hydrocolloids [280] or dietary fibers [281,282] to improve texture, and hence organoleptic properties. Building on knowledge derived from dairy applications and plant-based yogurt alternatives, more recent studies have investigated the influence of in situ EPS-producing starter cultures in the manufacture of products or base matrices suitable for plant-based cream cheese alternative development. Masiá, et al. [283] focused on the development of a plant-based cheese base matrix consisting of 10% pea protein isolate and varying concentrations of olive oil (0–20%). Matrix formation involved homogenization, heat pasteurization, cooling, and subsequent fermentation at 43 °C using the starter culture mix Vega™ Harmony (Chr. Hansen), composed of *Streptococcus thermophilus*, *Lactobacillus delbrueckii* subsp. *bulgaricus*, *L. acidophilus*, *Lacticaseibacillus paracasei*, and *Bifidobacterium animalis* subsp. *lactis*. Fermentation times required to reach a pH of 4.5 ranged from 5.5 to 7 h for all formulations and resulted in firm, particulate protein gels. These gels were characterized by a network of protein aggregates in which oil droplets were entrapped. Increasing oil concentrations hindered protein–protein interactions, leading to weaker gel structures. Although the authors did not focus specifically on spreadable products, but rather on potential plant-based cheese matrices in general, the results suggest that some of the matrices may serve as suitable base systems for the development of fermented plant-based cream cheese alternatives [284,285]. Beyond texture, the same research group examined flavor development related to plant-based cheese alternatives through the use of different culture blends for fermentation [286]. Shuai, et al. [287] investigated dextran-producing starter cultures from the *Weissella* and *Leuconostoc* genera for their ability to improve the texture of pea flour paste and analyzed the resulting microstructure. Dextran formed in situ during fermentation promoted a porous network that enhanced water-holding capacity and texture-related attributes. The texture-modulating effects were dominated by dextran–pea protein interactions, with dextran–starch interactions contributing to a lesser extent. A fermented cream cheese analog formulated from chickpeas and red lentil flour was investigated by Palatzidi, et al. [288]. Fermentation enhanced both its nutritional and its functional properties, resulting in products with enhanced textural properties, an altered amino acid profile (notably increasing levels of glutamate and glycine), and reduced antinutritional factors. Overall, these findings indicate that fermentation offers multiple advantages for improving the nutritional and organoleptic quality of plant-based cream cheese alternatives, and that fermentation with EPS-producing starter cultures provides a promising approach to further enhance the texture, as well as other quality attributes, of dairy analogs.

## 5. Conclusions

This review highlights both the opportunities and current challenges in developing plant-based dairy alternatives, with a particular focus on plant-based cream cheese alternatives. It considers not only product quality, but also formulation strategies informed by a comprehensive market analysis of existing products. Special attention is given to the potential of fermentation to enhance physicochemical and organoleptic attributes, especially texture, as well as nutritional properties. Furthermore, this review offers new perspectives on the role of EPS-producing starter cultures and identifies promising research directions in this domain. In addition, it explores how emerging technologies, such as PEF and US, may contribute to stimulating EPS production and modulating microbial activity. However, it is important to note that these technologies represent only a subset of available approaches. Other strategies, including process optimization, enzymatic treatments, or alternative processing technologies, may also play a significant role in improving plant-based dairy alternatives. Moreover, current research on the application of PEF and US in plant-based matrices remains limited, and further studies are required to evaluate their effectiveness under realistic processing conditions. Overall, future research should aim to systematically assess different technological approaches in complex plant-based systems, with particular emphasis on matrix-specific effects, scalability, and product quality.

## Data Availability

The datasets used and analyzed during the current study are available from the corresponding author on reasonable request.

## Appendix A. Methodology

### Appendix A.1. Literature Review

This article was designed as a comprehensive narrative review supported by a structured literature search. This review examines the potential of fermentation with exopolysaccharide (EPS)-producing lactic acid bacteria for the development of plant-based dairy alternatives, with particular emphasis on cream cheese alternatives and the potential of in situ-produced EPS to support texture development and reduce reliance on added hydrocolloids. In addition, it evaluates whether EPS production in these matrices could be stimulated through emerging technologies, specifically pulsed electric fields (PEF) and ultrasound (US). Literature was retrieved from scientific databases including Scopus, PubMed, Web of Science, Google Scholar, and Lirias, where applicable. Searches were performed using combinations of keywords related to four main thematic blocks: (i) fermentation with exopolysaccharide-producing lactic acid bacteria/starter cultures, (ii) EPS biosynthesis and in-matrix production, EPS structure, and techno-functional properties; (iii) acid-induced dairy products, plant-based dairy alternatives, cream cheese alternatives, role of proteins, hydrocolloids/stabilizers, fats, and fermentation/EPS; and (iv) PEF, US, and microbial stress responses. Reference lists of relevant articles were additionally screened to identify relevant publications not captured by the initial database searches. Due to the interdisciplinary scope of the topic and the limited number of studies e.g., directly addressing the use of emerging technologies to stimulate in situ EPS formation, no 5-year publication limit was applied. Instead, recent literature was prioritized, while older publications were also included when they were particularly relevant, foundational, or methodologically robust for explaining established mechanisms, technological principles, and matrix-related ingredient/EPS functionality relevant for possible knowledge transfer to plant-based dairy alternatives, particularly cream cheese alternatives.

Scope and Limitations: This article was designed as a narrative review with a structured literature search, rather than as a systematic review. Accordingly, it does not aim to provide a formal systematic evidence assessment, but rather a thematic synthesis of literature relevant to fermentation, EPS functionality, plant-based dairy matrices, and emerging technologies. Selection and interpretation of the literature were guided by relevance to the review objectives, scientific quality, and potential to support mechanistic understanding and knowledge transfer to fermented plant-based cream cheese alternatives.

### Appendix A.2. Market Study

To complement the literature review, a descriptive market study of commercially available plant-based cream cheese alternatives was conducted using available information obtained from online retailers (e.g., supermarket e-commerce websites), product websites, and the Innova Market Insights platform. Products were selected based on their market positioning as plant-based, vegan, or dairy-free alternatives to conventional cream cheese, including products labeled or described as cream cheese alternatives, spreads, fresh cheese alternatives, or similar products intended for comparable culinary use. The market survey primarily focused on Western European countries, including Austria, Belgium, France, Germany, Ireland, Switzerland, the Netherlands, and the UK; products and/or availability were also screened across other European countries, including Greece, Italy, Spain, Portugal, Finland, Norway, Sweden, the Czech Republic, Slovenia, and Latvia. In total, 201 plant-based cream cheese alternatives from 65 brands were evaluated, and information was collected on the used ingredients, including protein, fat, and hydrocolloid sources, as well as other ingredients or additives such as salt, acids, and preservatives. Declared nutritional composition was also recorded, as well as whether the product was fermented or contained a fermented ingredient.

Limitations: The analysis was based on publicly available product information (no laboratory verification of ingredient composition or nutritional values was performed). In addition, product availability, formulation, and labeling may vary between countries, retailers, and time points. The presented data reflect the market information available during the data collection period (mainly the end of 2023–2024) and may not capture all subsequent product (re)formulations or discontinuations. Accordingly, the data were interpreted as a large descriptive overview of market-relevant formulation trends, while acknowledging that product availability, formulations, and labeling may change over time, and were used to contextualize current formulation approaches relevant to the development of fermented (EPS-containing) vegan cream cheese alternatives.
